# Canopy plant composition and structure of Cape subtropical dune thicket are predicted by the levels of fire exposure

**DOI:** 10.7717/peerj.14310

**Published:** 2022-11-08

**Authors:** Tiaan Strydom, Tineke Kraaij, B. Adriaan Grobler, Richard M. Cowling

**Affiliations:** 1 Department of Conservation Management, Natural Resource Science and Management Cluster, Faculty of Science, Nelson Mandela University, George, Western Cape, South Africa; 2 African Centre for Coastal Palaeoscience, Nelson Mandela University, Gqeberha, Eastern Cape, South Africa

**Keywords:** Biome boundaries, Cape Floristic Region, Coastal dune vegetation, Forest, Fire frequency, Fynbos, Plant architecture, Species diversity, Structural composition

## Abstract

**Background:**

The subtropical dune thicket (hereafter “dune thicket”) of the Cape Floristic Region experiences a wide range of fire exposure throughout the landscape, unlike other dry rainforest formations that rarely experience fire. We sought to determine how fire exposure influences species composition and the architectural composition of dune thicket.

**Methods:**

We used multivariate analysis and diversity indices based on cover abundance of species to describe the species composition, architectural guild composition and structure of dune thicket sites subject to different levels of fire exposure, namely low (fire return interval of >100 years), moderate (fire return interval of 50–100 years), and high (fire return interval of 10–50 years).

**Results:**

The diversity, cover abundance and architectural guild cover abundance of dune thicket canopy species were strongly influenced by the level of fire exposure such that each level was associated with a well-circumscribed vegetation unit. Dune thickets subject to low fire exposure comprises a floristically distinct, low forest characterized by shrubs with one-to-few upright stems (ca. 4–8 m tall) and a relatively small canopy spread (vertical growers). Of the 25 species in this unit, 40% were restricted to it. Dune thickets subject to moderate fire exposure had the highest abundance of lateral spreaders, which are multi-stemmed (ca. 3–6 m tall) species with a large canopy spread and lower stature than vertical growers. None of the 17 species found in this unit was restricted to it. Dune thickets subject to high fire exposure had the highest abundance of hedge-forming shrubs, these being low shrubs (ca. 0.6–1.4 m tall), with numerous shoots arising from an extensive system of below-ground stems. Of the 20 species in this unit, 40% were restricted to it. Multivariate analysis identified three floristic units corresponding to the three fire exposure regimes. Compositional structure, in terms of species and architectural guilds, was most distinctive for dune thickets subject to high and low fire exposure, while the dune thicket subject to moderate fire exposure showed greatest compositional overlap with the other units.

**Conclusion:**

Fire exposure profoundly influenced the composition and structure of dune thicket canopy species in the Cape Floristic Region. In the prolonged absence of fire, the thicket is invaded by vertical-growing species that overtop and outcompete the multi-stemmed, laterally-spreading shrubs that dominate this community. Regular exposure to fire selects for traits that enable thicket species to rapidly compete for canopy cover post-fire *via* the prolific production of resprouts from basal buds below- and above-ground. The trade-off is that plant height is constrained, as proportionately more resources are allocated to below-ground biomass.

## Introduction

Climatically and edaphically homogeneous landscapes may support structurally and floristically distinct biomes, raising the question as to what determines the boundaries between them (Bond, Midgley & Woodward, 2003; Hoffmann et al., 2012; Butler et al., 2014; Coetzee, Bond & Wigley, 2015; Cowling & Potts, 2015; Cramer et al., 2019). In landscapes dominated by fire-prone vegetation such as savannas, grasslands and heathlands, fire regime effects have been invoked to explain the boundaries between fire-prone and fire-avoiding biomes, for example Afrotemperate forest patches in Cape fynbos shrublands (Manders, 1990; Geldenhuys, 1994; Cowling & Potts, 2015; Bond, Midgley & Woodward, 2003) and rainforest patches in savanna and grassland (Hoffmann et al., 2009; Murphy & Bowman, 2012; Becket et al., 2022). The coastal dunes of the Cape Floristic Region (CFR) are an interesting case in this regard, since they can support three biomes, namely dune forest, subtropical dune thicket (hereafter “dune thicket”) and dune fynbos, each of which experiences different fire regimes as determined by topographically-induced fire protection and species-specific flammability properties (Pierce & Cowling, 1991; Cowling & Potts, 2015; Msweli et al., 2020; Cowling & Hoffman, 2021). Here we report on the compositional and architectural differences of thicket canopy dominants in dune communities associated with different levels of fire exposure. We investigated whether the historical fire frequencies purported for these communities’ influence dune thicket composition and structure.

The effects of fire regimes on vegetation composition and structure have been studied extensively in fire-prone vegetation across the globe (*e.g.*, Bond & van Wilgen, 1996; Williams, Gill & Bradstock, 2012; Rundel et al., 2018; Becket et al., 2022). Even though dune thicket is not a fire-dependent vegetation type, it experiences fire and is resilient to high fire severity as most, if not all, thicket canopy species are capable of resprouting post-fire from dormant basal buds or epicormic buds in the canopy (Cowling & Pierce, 1988; Strydom et al., 2020). Strydom et al. (2020) found no evidence of a relationship between fire severity and thicket shrub survival. However, a few single-stemmed tree species (*Apodytes dimidiata* E.Mey. ex Arn., *Chionanthus foveolatus* (E.Mey.) Stearn, *Scolopia zeyheri* Szyszyl.), which mostly grow in deep, narrow dune swales where community composition and structure are more typical of dune forest than of thicket (Cowling, 1984; Cowling et al., 1997; Midgley et al., 1997), are more vulnerable to high fire severity, displaying lower survival rates and weaker resprouting vigour than species which have two or more stems at ground level (*e.g., Pterocelastrus tricuspidatus* Walp., *Searsia glauca* (Thunb.) Moffett, *Sideroxylon inerme* Forssk) (Strydom et al., 2020, 2021). Furthermore, a largely positive relationship between tree size and fire survival suggested that some species of dune thicket may be negatively affected by frequent fires (Strydom et al., 2020).

The long-term effects of fire severity and frequency on species composition and structure of dune thicket are, however, not known. Strydom et al. (2021) classified thicket species into architectural guilds and post-fire resprouting guilds, which could be used in the current study to assess structural composition of dune thicket in relation to long-term fire exposure. Three architectural guilds were recognised, namely vertical growers (tall-stature (ca. >4 m), single-stemmed trees with a small to large canopy spread), lateral spreaders (moderate-stature (ca. 2–4 m), multi-stemmed shrubs with a large canopy spread), and hedge formers (low-stature, (ca. <2 m) multi-stemmed shrubs with a large canopy spread). Hedge-forming species exhibited a high mean survival rate (>80%) and strong resprouting vigour post-fire; lateral-spreading species also exhibited a high mean survival rate (>80%) and moderate to strong resprouting vigour post-fire; whereas vertical-growing species showed a lower mean survival rate (73%) and weaker resprouting vigour post-fire (Strydom et al., 2020, 2021).

Several studies have investigated the influence and importance of fire on species composition and structure of savanna-forest mosaics of Africa, Australia and Brazil (Charles-Dominique et al., 2015; Hoffmann et al., 2009; Murphy & Bowman, 2012; Flake et al., 2021a; Flake et al., 2021b; Becket et al., 2022). In these systems, regular fires promote savanna tree and grass abundance and suppress forest trees, which are mostly killed by fire; however, when the fire frequency increases, savanna trees can also be suppressed, thereby favoring grasslands. Accordingly, if fire is absent for too long, forest tree abundance will increase, and savanna tree and grass cover will decrease (Hoffmann et al., 2009; Flake et al., 2021b; Becket et al., 2022). Less is known about the influence of fire on species composition and structure of closed-canopy vegetation types that rarely burn, such as Afrotemperate forest (Watson & Cameron, 2001; Giddey, Baard & Kraaij, 2022a) and thicket (Cowling, 1984; Vlok, Euston-Brown & Cowling, 2003; Cowling & Potts, 2015); however, some information on species-specific responses to fire is available, which leads to the question of how fire exposure shapes community composition in these closed communities.

Forest margins in forest-fynbos mosaics in the CFR burn to a greater or lesser extent (depending on the fynbos fuel load and the severity of fire weather conditions) when the adjacent fynbos burns (Giddey et al., 2021). The level of fire exposure that forest patches experience depend on their position in the landscape in relation to topography and desiccating winds that drive fires (Geldenhuys, 1994; Cowling & Potts, 2015). Forest margins and small forest patches typically experience more frequent and severe fire than the forest core and large patches (Geldenhuys, 1994; Giddey, Baard & Kraaij, 2022a; Giddey, Baard & Kraaij, 2022b). Like the forest core, the dune thicket core is seldom exposed to fire and will only burn under the most severe conditions, likely less than once per century. The margins of dune thicket patches and small thicket clumps, on the other hand, may burn whenever fire occurs in the dune fynbos matrix, typically every 10–20 years (Cowling et al., 1997; van Wilgen et al., 2010; Kraaij et al., 2013).

Watson & Cameron (2001) investigated the influence of fire on Afrotemperate forest composition and found tree diversity to be higher in the fire-sheltered forest core, which contained a unique suite of species that were absent from the fire-exposed forest margin. They found that the young trees of *Gonioma kamassi* E.May, *Ilex mitis* (L.) Radlk., and *Podocarpus latifolius* hort. ex Carrière. that occurred in the forest margin had high mortality rates (75–100%) post-fire, suggesting that fire-induced mortality can lead to a change in species composition. Similarly, Giddey, Baard & Kraaij (2022b) found that Afrotemperate forest tree species showed differential post-fire survival responses, although some individuals of virtually all species were able to survive fire by resprouting. Certain species, *e.g*., *Olea capensis* subsp. *macrocarpa* (C.H.Wright) I. Verd, *Podocarpus latifolius*, *Elaeodendron croceum* DC. and *Halleria lucida* L. showed high mortality rates (>60%) whereas others, like *Ocotea bullata* (Burch.) Baill., *Lachnostylis hirta* Mǜll.Arg., *Cassine peragua* L. and *Rapanea melanophloeos* Mez, showed low mortality rates (<40%) and strong resprouting capabilities. Like forest trees, single-stemmed vertical-growing species in dune thicket, *i.e., Chionanthus foveolatus, Apodytes dimidiata* and *Scolopia zeyheri*, had higher mortality rates post-fire (40–60%) compared to multi-stemmed lateral spreaders *e.g., Mystroxylon aethiopicum* (14%), *Pterocelastrus tricuspidatus* (10%) and hedge forming species *Searsia glauca* (19%) (Strydom et al., 2020).

Given that dune thicket shrub species have variable post-fire survival rates, resprouting abilities and architecture (Strydom et al., 2020; Strydom et al., 2021), and need to compete for light and belowground resources in a post-fire environment, we asked the question: How does fire exposure influence the species composition and structure (~architectural guild composition) of dune thicket in the same landscape? Based on the ecologies of the respective architectural guilds, it should be possible to predict their occurrence in relation to gradients of fire exposure. We also expect differences in floristic composition to parallel the differences in architectural guild composition in relation to fire exposure. While there are some data consistent with these predictions (Cowling, 1984; Cowling et al., 1997; Midgley et al., 1997), they remain to be rigorously tested. Thus, we investigated whether the degree of fire exposure influences species composition and architectural guild (*sensu* Strydom et al., 2021) composition of dune thicket canopy species in a coastal dune landscape.

## Methods

### Study area

**Figure 1:**
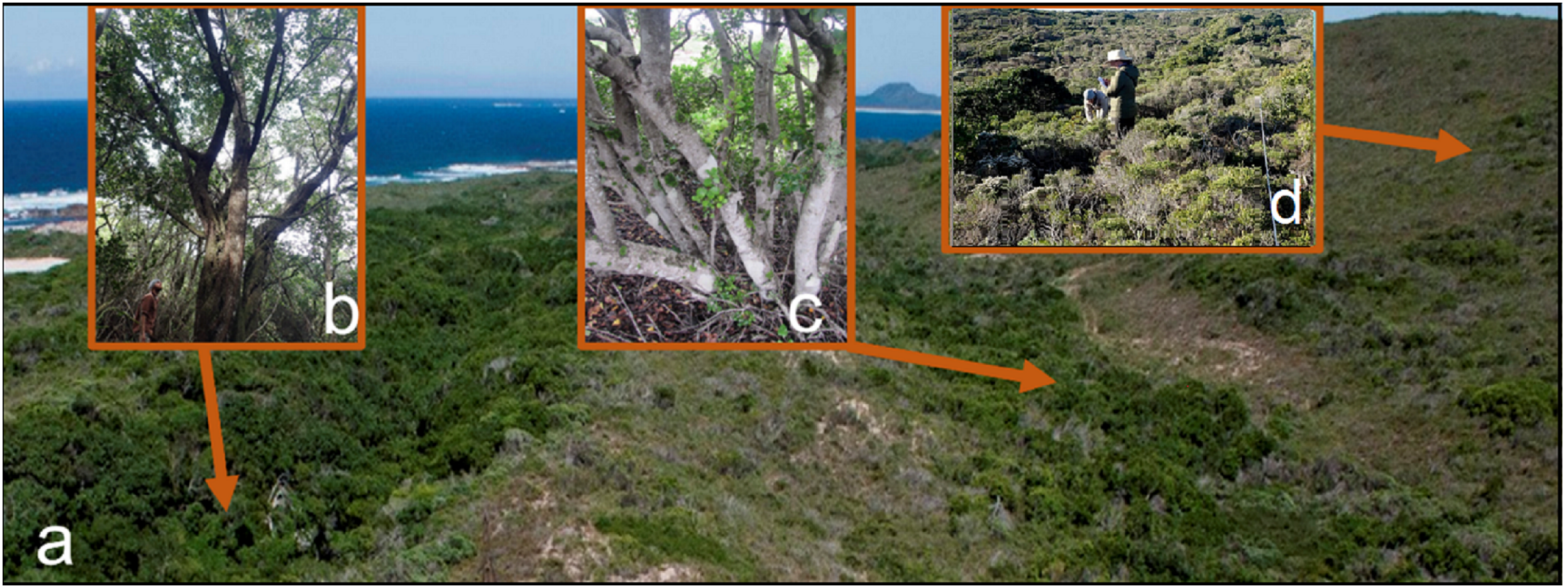
Fire exposure and associated thicket structure of the dune landscape. (A) Dune landscape of the study area showing parallel dune ridges and associated swales. (B) Narrow and steep-sided dune swale that is seldom, if ever, exposed to fire and supports ca. 4–8 m-tall dune forest. (C) Broad and shallow dune swale that supports ca. 3–6 m-tall dune thicket that only burns in the most severe fires at intervals of ca. 50–100 years. (D) Dune crests and slopes that supports ca. 0.6–1.4 m-tall dune fynbos-thicket that are exposed to fires at intervals of ca. 10–50 years.

The study was conducted in a landscape comprising parallel ridges and associated swales of coastal dunes west of Cape St Francis in the southeastern CFR (Fig. 1). The geology comprises Holocene aeolianites (Schelmhoek Formation) overlying Pleistocene calcarenites (possibly Nahoon Formation) (Roberts et al., 2006; Cowling et al., 2019). Soils are mostly deep, calcareous sands with high organic matter content (Cowling, 1984). The climate is warm-temperate, and rainfall occurs year-round but with relatively dry summers. The mean annual rainfall for the region is ca. 700 mm. The mean annual temperature is 17 °C with a minimum of 4 °C and maximum of 32 °C, with the warmest months being December-February and the coldest being July–August. The wind regime is fierce, with east to southeasterly winds dominating in summer, and west to southwesterly winds in winter; gale force winds occur mainly in the spring and summer months.

The vegetation of the study area comprises a mosaic of thicket and fynbos vegetation, with scattered and small (1–5 ha) patches of forest (Cowling, 1984; Cowling et al., 2019). Both thicket and forest canopy species produce bird-dispersed propagules that established in shaded microsites (Cowling et al., 1997; Strydom et al., 2019). The understorey vegetation comprises a relatively depauperate flora of forbs and graminoids (Cowling, 1984). The herb and graminoid layer in these dune thickets are not well developed and comprise only a few species when present (Cowling, 1984). Fynbos, which invariably has an admixture of low thicket shrubs, is confined largely to the well-drained slopes and crests of the hairpin parabolic ridges that run in an east to west direction, which aligns with the prevailing wind directions (Fig. 1). Thicket is mainly associated with the lower slopes and swales of the dune landscape; here, owing to the presence of an impermeable layer (calcarnite or quartzitic sandstone) near the soil surface, soil moisture is more accessible to plant roots than on the deeper soils of the dune ridges. The small patches of forest are restricted to narrow dune swales with steep-sided dune ridges. Soil chemical and physical variables are similar across the landscape except for organic carbon, which is higher in forest than thicket or fynbos.

The grazing/browsing regime in the study has changed in the past several years. Prior to the colonial era (starting ca. 1750), the area experienced episodic grazing by cattle and sheep belonging to nomadic Khoe-khoe pastoralists (Cowling, 1984) as well as browsing by indigenous herbivores including two megaherbivores (Boshoff & Kerley, 2001; Radloff, 2008). The colonial era saw the extirpation of megaherbivores and increase in grazing of livestock belonging to sedentary pastoralists of European origin alongside browsing by small medium-sized indigenous herbivores. Since the early 1960s, grazing domestic livestock has declined and herbivory is now entirely by small to medium-sized indigenous herbivores.

The dune topography of the study area (Fig. 1) influences the potential occurrence of fire in the landscape (Cowling, 1984; Cowling & Hoffman, 2021). Dune ridges and slopes, comprising highly flammable fynbos (Cowling, 1984; Calitz, Potts & Cowling, 2015; Msweli et al., 2020), are exposed to the full brunt of the region’s strong wind regime; these sites likely burn in every conflagration. The thicket-dominated dune-swales, comprising species of lower flammability (Cowling, 1984; Calitz, Potts & Cowling, 2015; Msweli et al., 2020), and subject to a milder wind regime than the slopes, burn only under extreme fire hazard conditions. The deep, narrow swales that support forest are protected from fire, even under the most severe conditions, as were experienced in a wildfire in the summer of 2016.

In the absence of detailed fire records, we identified a fire exposure gradient in the dune landscape in relation to topographical position and presumed historical occurrence of fire as gleaned from 10 aerial photographs (1961, 1969, 1985, 2000, 2006, 2009, 2011, 2013, 2016, 2019) (Fig. S1) (CDNGI Geospatial Portal V 16.5.0105 Build: 00054, 2022; Google Earth Pro V 7.3.4.8573, 2022), repeat photography (Cowling & Hoffman, 2021), and anecdotal accounts (Table S1) from long-term residents in the area of the occurrence of wildfires since the 1930s. Note that the regime for the dune fynbos-thicket mosaic is likely biased towards lower fire frequencies owing to fire suppression practices initiated with a shift from pastoralism to resort development in the early 1960s. The aerial photographs and anecdotal accounts of fire occurrence confirmed that the distribution of dune forest was stable and had not burnt since 1942. Thicket appears to have expanded into fynbos in the dune swales and lower slopes over the same period but especially since the early 1960s, a finding that supported by analysis of repeat photography (Cowling & Hoffman, 2021).

The study area experienced a fire in January 2016, which burnt large parts of dune fynbos and thicket on slopes and broad swales (Strydom et al., 2020), leaving the fire-protected forest of the deep, narrow swales unburnt. The fairly crude estimations of fire occurrence revealed by these sources enabled us to categorize fire exposure in terms of three broad categories: (i) low fire exposure (*i.e.*, fire return intervals of >100 years) associated with deep, narrow dune swales (Fig. 1) that provide refuge from strong winds and wind-driven fires, and which support dune forest vegetation; (ii) moderate fire exposure (*i.e.*, fire return intervals of 50–100 years) associated with dune swales, that experience moderate exposure to wind, and which support dune thicket; and (iii) high fire exposure (*i.e.*, fire return intervals of 10–50 years) associated with dune crests and upper slopes that experience the strongest wind and fire exposure in the dune landscape, and which support a dune fynbos-thicket mosaic comprising ericoid shrubs, restioids and dwarf thicket shrubs.

### Data collection

During April 2018, we surveyed 17 belt transects in each of the three dune thicket fire exposure categories, focusing on native canopy-forming shrubs and trees, but excluding fynbos shrubs in the high fire exposure sites (taxonomic authorities for all study species are listed in Table 1). Alien trees were absent from our transects. We used transects of 50 m × 5 m in low and moderate fire-exposed sites. We surveyed the latter, which burnt in 2016, at a post-fire age of almost 3 years by measuring the burnt shrub skeletons which resembled the architecture of the mature pre-fire individuals. In high fire-exposed sites we surveyed 17 belt transects of 5 m × 2 m. The smaller transect size used here was due to extreme stem density of the dwarf dune thicket shrubs (mean density of 5.2 stems/m^2^ *vs*. 0.4 stems/m^2^ in moderate and low fire exposure). These transect sizes produced reasonably comparable survey effort across transects, *i.e.*, the mean number of stems assessed per transect was 95 in low fire exposure, 73 in moderate, and 52 in high fire exposure. Variation in slope, aspect and soil type (Cowling, 1984; Cowling & Potts, 2015) of the surveyed transects were broadly comparable amongst the different fire exposure sites (Table S2). In sites associated with low and moderate fire exposure, individuals with a stem diameter >5 cm were recorded and all stems that were connected at the base at ground level were considered an individual. In high fire exposure no threshold was set as individual shrubs have small stems and all stems that were connected at the base at ground level were considered an individual. In all fire exposure categories individual plant canopy height and the widest and shortest canopy diameter were measured to estimate cover abundance of species and architectural guilds of canopy shrubs (detailed below).

**Table 1:** Composition, canopy cover abundance and architectural guild allocation (Strydom et al., 2021) of canopy species in three fire exposure categories (low, moderate, high). Architectural guilds included vertical grower (V), lateral spreader (L), and hedge former (H). Species allocated to more than one guild showed architectural plasticity across fire exposure categories.

	Projected canopy cover (%)		
Species	Low fire exposure	Moderate fire exposure	High fire exposure
			
*Canthium spinosum* (L.f.) J.St.-Hill.	6.8 (V)		
*Chionanthus foveolatus* E.Mey. Stearn	4.2 (V)		
*Allophylus decipiens* Radlk.	1.4 (V)		
*Apodytes dimidiata* E.Mey. ex Am.	1.4 (V)		
*Scolopia mundii* Warb.	0.5 (V)		
*Schotia afra* Thunb.	0.2 (V)		
*Acokanthera oppositifolia* (Lam.) Codd	0.1 (V)		
*Gymnosporia nemorosa* Szyszyl.	0.1 (V)		
*Celtis africana* Burm.f.	0.04 (V)		
*Dovyalis rhamnoides* Engl.	11.5 (V)	0.1 (V)	
*Psydrax obovata* Bridson	6.3 (V)	0.8 (V)	
*Scolopia zeyheri* Warb.	5.1 (V)	0.6 (V)	
*Zanthoxylum capense* Harv.	3 (V)	1.3 (V)	
*Clausena anisata* Hook.f., De Wild. & Staner	1.0 (V)	0.04 (V)	
*Gymnosporia buxifolia* (L.) Szyszyl	0.3 (L)	0.1 (L)	
*Mystroxylon aethiopicum* (Thunb.) Loes.	33.4 (L)	2.9 (L)	0.02 (H)
*Pterocelastrus tricuspidatus* Walp.	30.9 (L,V)	50.2 (L,V)	2.5 (L,H)
*Sideroxylon inerme* Forssk.*^#^*	25.5 (L,V)	6.1 (L,V)	8.5 (L,H)
*Cassine peragua* L.	5.2 (V)	0.6 (V)	0.2 (H)
*Scutia myrtina* (Burm.f.) Merr.	1.7 (H)	0.6 (H)	0.1 (H)
*Dovyalis rotundifolia* Engl.	1.3 (V)	0.4 (V)	0.3 (H)
*Searsia glauca* (Thunb.) Moffett.	3.0 (H)	7.7 (H)	22.9 (H)
*Euclea racemosa* L.*	0.8 (V)	4.7 (V)	2.7 (H)
*Olea exasperata* Jacq.^+,^*	0.5 (V)	0.01 (H)	28.7 (H)
*Osyris compressa* A.DC.	1.0 (L)		3.1 (H)
*Searsia lucida* (L.) F.A.Barkley.		1.7 (H)	3.8 (H)
*Searsia crenata* (Thunb.) Moffett^+,^*		0.02 (H)	1.1 (H)
*Maytenus procumbens* (L.f.) Loes.			10.7 (H)
*Robsonodendron maritimum* (Bolus) R.H.Archer^+^			7.9 (H)
*Rapanea gilliana* Mez^@,+,^*			3.0 (H)
*Lauridia tetragona* (L.f.) R.H. Archer			1.6 (H)
*Carissa bispinosa* (L.) Merxm.			1.5 (H)
*Putterlickia pyracantha* (L.) Szyszyl.			1.5 (H)
*Cussonia thyrsiflora* Thunb.			1.2 (H)
*Searsia laevigata* (Moffett) Moffett.*			0.1 (H)

**Notes:**

# Protected.

@ Endangered.

+ Cape dune endemic.

* Geoxyle (recruit from below ground stems).

Most thicket species are classified as least concerned on the IUCN red list of threatened species (IUCN, 2022) but one species is classified as endangered (Victor, 2006). One species are protected by law (South African Government, 2022) and a few species are endemic (Cowling et al., 2019).

### Data analysis

We used the Sørensen similarity coefficient to assess the similarity in species composition of the different fire exposure categories in terms of the proportion of shared species pooled across all transects within the respective fire exposure categories (C_s_ = 2 × *c*/*S*_1_ + *S*_2_) (c = number of species in common between both communities, S_1_ = number of species in community one; S_2_ = number of species in community two) (Sørenson, 1948). We used projected canopy cover (%) to estimate cover abundance of dune thicket species (rather than numbers of individuals per species, due to difficulty with identifying individuals in the case of clonal plants) and architectural guilds in all fire exposure categories. Canopy cover can exceed 100% as dune thicket shrub canopies often overlap. The canopy area (m^2^) of each individual shrub was calculated as the area of an oval (a/2 × b/2 × π) where a = widest canopy diameter, and b = shortest canopy diameter. The total canopy area (m^2^) for each species per transect was calculated as the sum of the canopy area of all the individuals of that species within the transect. To calculate % canopy cover of each species per transect we divided the total canopy area of the species by the area of the transect and multiplied by 100.

To calculate the % canopy cover per architectural guild for each transect, we assigned each species, based on its observed architecture in each transect and fire exposure category, to an architectural guild (*i.e.*, hedge former, lateral spreader, or vertical grower). This categorisation was based on the physical dimensions associated with each architectural guild as defined by Strydom et al. (2021). The total canopy areas of all species assigned to an architectural guild were summed to obtain the total canopy area of each guild per transect. Thereafter the % canopy cover was calculated for each architectural guild by dividing with the area of the transect and multiplying by 100.

We calculated species diversity (with species data pooled across transects within fire exposure categories) using Shannon-Wiener index (Spellerberg & Fedor, 2003) and did pairwise comparisons between the three fire exposure categories using Hutcheson t-test (Hutcheson, 1970). Transects were used as the unit of replication in all subsequent analyses.

Canopy cover data were normally distributed, and a one-way ANOVA followed by a *post-hoc* (Tukey) test was used to compare canopy cover (which indicates canopy overlap when exceeding 100%) between the fire exposure categories. Canopy height data were not normally distributed and a Kruskal-Wallis test followed by a *post-hoc* (Dunn’s) test were used to compare canopy height between the fire exposure categories.

Although our primary interest was in the effect of fire exposure on species and structural composition, we used distance-based Redundancy Analysis (db-RDA) (using the ‘dbrda’ function from the ‘vegan’ version 2.5-7 R package; Oksanen et al., 2020), with fire exposure, slope and aspect as environmental factors, to assess the potential influence of these factors on dune thicket composition. These, and all subsequent analyses, were conducted in R version 4.1.1 (R Core Team, 2021). Prior to db-RDA, the species and architectural-guild cover abundance data were square root transformed and subjected to Wisconsin double standardisation, after which Bray-Curtis dissimilarity matrices were compiled for use in subsequent analyses. We then constructed maximal db-RDA models (*i.e*., constrained by all environmental factors) and used the ‘cca.anova’ function to assess the significance of the environmental factors’ marginal effects on species and structural composition through permutational tests (999 permutations). In the following steps, we only included factors that had significant marginal effects in the final db-RDA of guilds and species (*i.e*., those used for ordination plots). As our focus was on the effect of fire exposure, where factors other than fire exposure were found to have significant marginal effects on composition, we used partial db-RDA to control for these factors.

Next, we used cluster analysis (CA) and ordinations based on db-RDA (see details above) and non-metric multidimensional scaling (NMDS) to explore the effect of fire exposure on patterns of species and architectural guild cover abundance among the 51 transects spread across the three fire exposure categories. Our hierarchical CA was based on a Bray-Curtis dissimilarity matrix and used Ward’s agglomeration method (‘ward.D’ option in the ‘hclust’ function). The NMDS analysis was implemented *via* the ‘metaMDS’ function of the ‘vegan’ version 2.5-7 R package (Oksanen et al., 2020), which has incorporated various procedures to facilitate a robust solution (Minchin, 1987). Cover abundance data were square root transformed and subjected to Wisconsin double standardisation, after which a Bray-Curtis dissimilarity matrix was used to ordinate the data. The ordination was run 999 times with random starts to prevent the NMDS from becoming trapped in local optima, and the solution with minimal stress was then selected. To facilitate interpretation of the results, the final NMDS solution was centred and rotated by principal components so that the variance of points was maximised along the first NMDS axis. We further used permutational multivariate analysis of variance (PERMANOVA), implemented *via* the ‘adonis’ function of the ‘vegan’ version 2.5-7 R package (Oksanen et al., 2020), to test for differences in cover abundances of species and architectural guilds among the fire exposure categories. The ‘pairwiseAdonis’ version 0.4 R package (Martinez Arbizu, 2017) was then used for *post-hoc* multilevel pairwise comparisons between the three fire exposure categories. For further investigation of species abundance across fire exposure categories we used a rank abundance curve. The code for NMDS, CA, and db-RDA analysis are provided here: https://zenodo.org/record/7133190#.YzhDIHZBw2w.

## Results

### Species diversity and composition

A total of 35 thicket shrub and low tree species were recorded in the vegetation survey (Table 1). Vegetation experiencing low fire exposure comprised 25 species, of which nine were exclusive to this category. Vegetation experiencing moderate fire exposure comprised 17 species, none of which were exclusive to this category. Vegetation experiencing high fire exposure comprised 20 species of which eight species were exclusive to this category. Nine species were shared among all fire exposure categories (Table 1; Fig S5).

Species with the highest cover abundance in low fire-exposed sites were *Mystroxylon aethiopicum*, followed by *Pterocelastrus tricuspidatus, Sideroxylon inerme* and *Dovyalis rhamnoides*. In moderate fire-exposed sites, the most abundant species was *Pterocelastrus tricuspidatus*, followed by *Searsia glauca, Sideroxylon inerme* and *Mystroxylon aethiopicum*. In high fire-exposed sites, the dominant species was *Olea exasperata*, followed by *Searsia glauca, Maytenus procumbens* and *Sideroxylon inerme*. Low fire-exposed sites supported several forest species, namely *Apodytes dimidiata, Chionanthus foveolatus, Scolopia mundii, Psydrax obovata* and *Celtis africana*. High fire-exposed sites were the exclusive habitat for several dune-endemic thicket species *e.g*., *Cussonia thyrsiflora, Rapanea gilliana, Robsonodendron maritimum* and *Searsia crenata*. Nine species that occurred across all fire exposure sites showed consistent differences in canopy coverage in relation to them (Fig. 1; Table 1; Fig. S2).

The Sørenson similarity coefficient (Ss) showed higher compositional similarity between low and moderate fire exposure categories (Ss = 71%) than between low and high fire exposure (Ss = 40%), or between moderate and high fire exposure (Ss = 60%). The Shannon-Wiener diversity index value (H) was significantly lower for sites subject to moderate fire exposure (H = 1.3) than for those subject to low fire exposure (H = 2.3; t = 5.76, *P* < 0.001) and to high fire exposure (H = 2.2; t = 4.90; *P* = 0.001), while it did not differ between low and high fire exposure sites (t = 0.32, *P* = 0.76).

Permutational significance tests of the maximal db-RDA model (Table 2) confirmed that slope was not a significant predictor (*P* = 0.190) of dune thicket species composition, but aspect did have a marginal effect (*P* = 0.031), which was mostly associated with variation in species composition within fire exposure groups, especially within high fire exposure, rather than between fire exposure groups (Fig. S3). Fire exposure had the most significant marginal effect on species composition of dune thicket (*P* = 0.001).

**Table 2:** (A) Permutational significance test for the maximal distance-based redundancy analysis model based on dune thicket species cover abundance. (B) Permutational significance tests of marginal effects of environmental factors specified in the maximal distance-based redundancy analysis model based on dune thicket species cover abundance.

	Df	SumsOfSqs	*F*-value	*P*-value
A				
Model	9	7.5103	4.29544	0.001
Residual	41	7.9652		
B				
Fire exposure	2	5.0028	12.8757	0.001
Slope	3	0.7500	1.2868	0.190
Aspect	4	1.1524	1.4829	0.031
Residual	41	7.9652		

**Figure 2:**
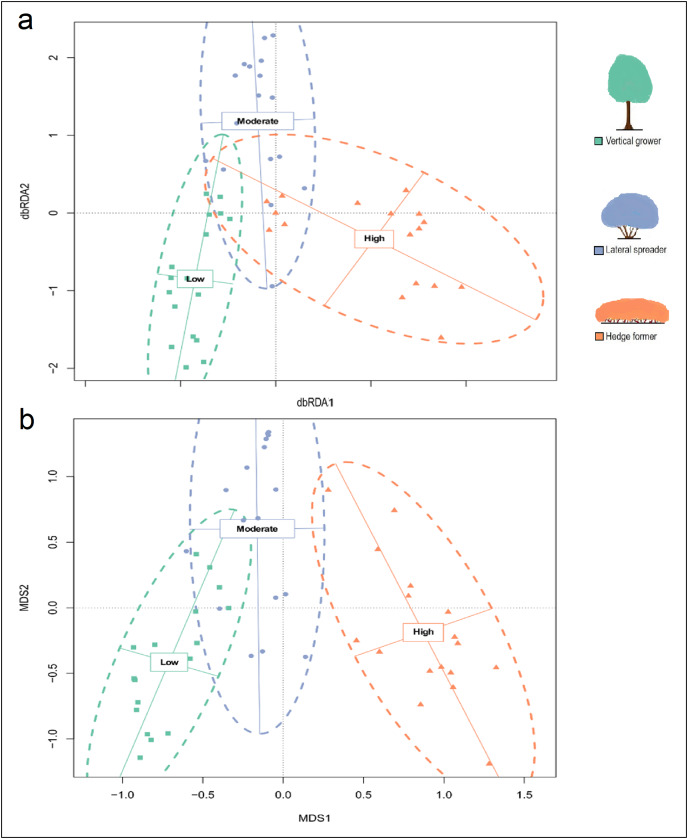
(A) Partial distance-based redundancy analysis (db-RDA) ordination plot (constrained by fire exposure) and (B) mutlidimensional scaling (MDS) ordination plot (uconstrained) for cover abundance data of dune thicket species. The db-RDA and MDS ordinations were plotted using a Bray-Curtis dissimilarity matrix based on cover abundance data of species and projected onto two-dimensional space. The shapes (square, circle, triangle) are transects colored according to their location in pre-determined fire exposure categories for dune thicket (green = low fire exposure; blue = moderate fire exposure; orange = high fire exposure). The dashed ellipses indicate 95% confidence intervals around the centroids of each of these categories. The effect of aspect has been controlled for in this dbRDA. The legend (color coded) shows the architectural guild which is most common in the respective fire exposure categories.

The partial db-RDA (effect of aspect controlled for), hierarchical CA and NMDS ordinations based on species abundances identified three dune thicket units that aligned with our fire exposure categories (Fig. 2; Figs. S4 and S5). These ordinations illustrated the dissimilarities between the fire exposure categories in terms of the species’ cover abundances in the transects. There were some similarities between certain transects of moderate fire exposure and those of low and high fire exposure, but most overlap occurred between transects of low and moderate fire exposure. The similarities between transects of low and moderate fire exposure were a result of similar cover abundances of shared species, such as *Pterocelastrus tricuspidatus*, *Sideroxylon inerme*, *Mystroxylon aethiopicum*, and *Dovyalis rotundifolia*. PERMANOVA (*F* = 15.051, *P* = 0.001) and *post-hoc* pairwise multi-level comparison (adjusted *P* = 0.003 for all comparisons) showed that there were significant differences in the cover abundances of species between all three fire exposure categories (Table S3). These differences were accounted for by species that showed high fidelity to sites of either low or high fire exposure. Overall, sites subject to moderate fire exposure had the lowest compositional distinctiveness and the lowest species diversity whereas sites in low and high fire exposure had distinctive floras of similarly high diversity.

### Architectural guild composition

**Figure 3:**
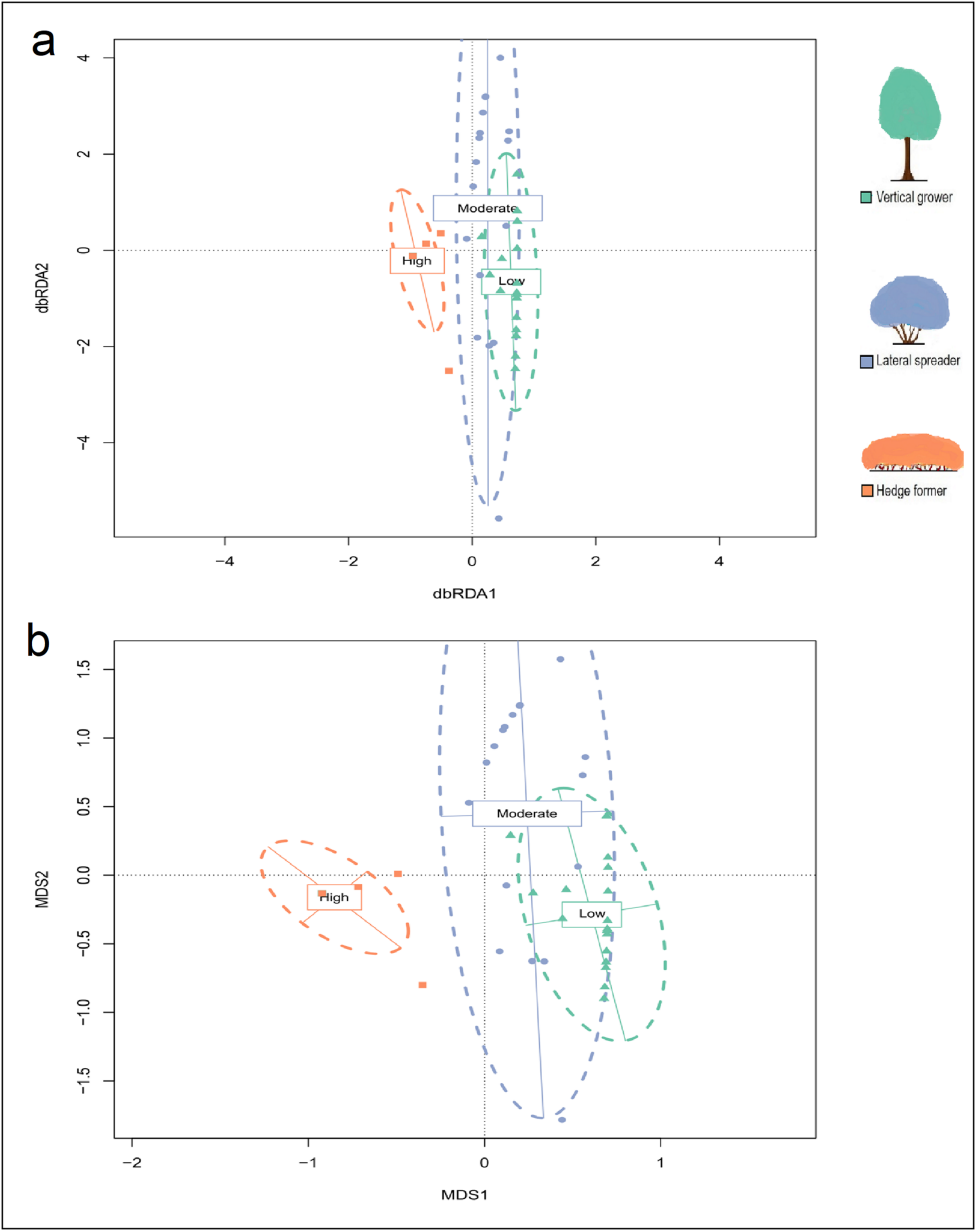
Distance-based Redundancy Analysis (db-RDA) ordination plot (constrained by fire exposure) and (B) Mutlidimensional scaling (MDS) ordination plot (unconstrained) for cover abundance data of dune thicket architectural guilds. The db-RDA and MDS ordinations were plotted using Bray-Curtis dissimilarity matrix based on cover abundance data of architectural guilds and projected onto two-dimensional space. The shapes (square, circle, triangle) are transects colored according to their location in pre-determined fire exposure categories for dune thicket (green = low fire exposure; blue = moderate fire exposure; orange = high fire exposure). The dashed ellipses indicate 95% confidence intervals around the centroids of each of these categories. The legend (color coded) shows the architectural guild which is most common in the respective fire exposure categories.

**Figure 4:**
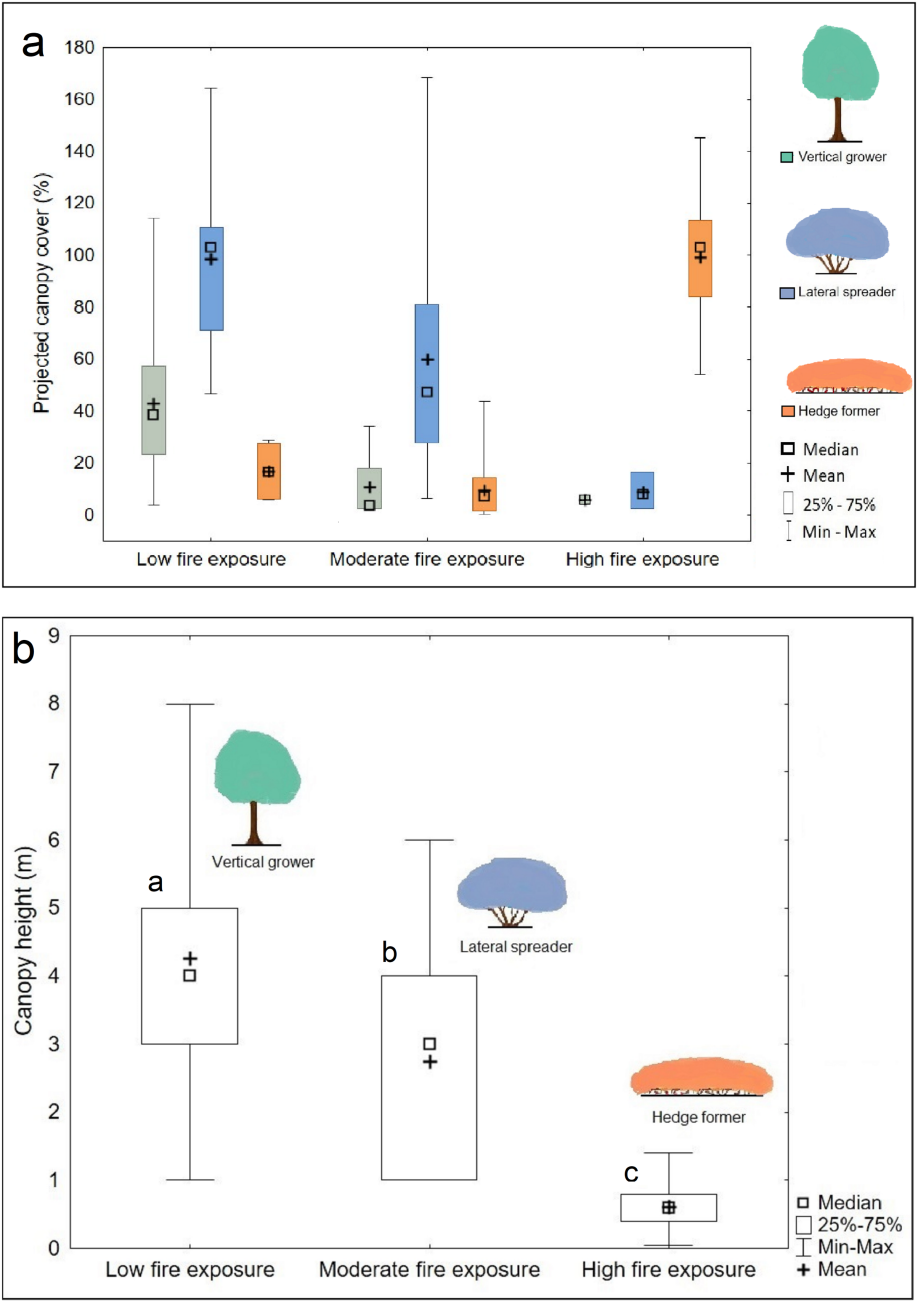
Canopy cover (A) and height (B) differences across three fire exposure categories. Note that canopy cover values may exceed 100% owing to canopy overlap. The legend (color coded) shows the architectural guild which is most common in the respective fire exposure categories. Disparate small letters denote significant differences among fire exposure categories based on Kruskal Wallis *H* test results and Dunn’s multiple comparisons.

Analysis of the maximal db-RDA model (Table 3) showed that slope (*P* = 0.917) and aspect (*P* = 0.350) did not have significant marginal effects on the structural composition of dune thicket; however, the level of fire exposure did (*P* = 0.001). The db-RDA, hierarchical CA and NMDS ordinations of architectural guild cover identified three units that aligned with the three fire exposure categories, but with a stronger overlap between low and moderate fire exposure than was the case for species composition (Fig. 3; Figs. S6 and S7). There were similarities between certain transects of low and moderate fire exposure, showing similar cover abundance in architectural guilds, especially in terms of lateral spreaders (Fig. 4A, Fig. S7). The structural homogeneity of dune thicket in high fire exposure settings was pronounced as 14 of the 17 transects – all comprising exclusively hedge-forming shrubs – were indistinguishable in the db-RDA and NMDS ordinations. PERMANOVA (*F* = 59.237, *P* = 0.001) and *post-hoc* pairwise multi-level comparison (adjusted *P* = 0.003 for all comparisons) showed that there were significant differences between the architectural guild cover abundances of the three fire exposure categories (Table S4*)*.

**Table 3:** (A) Permutational significance test for the maximal distance-based redundancy analysis model based on dune thicket architectural guild cover abundance. (B) Permutational significance tests of marginal effects of environmental factors specified in the maximal distance-based redundancy analysis model based on dune thicket architectural guilds cover abundance.

	Df	SumsOfSqs	*F*-value	*P*-value
A				
Model	9	7.8110	30.645	0.001
Residual	41	1.1612		
B				
Fire exposure	2	6.2887	111.0247	0.001
Slope	3	0.0200	0.2355	0.917
Aspect	4	0.1372	1.2109	0.350
Residual	41	1.1612		

The hedge-forming guild dominated high fire exposure sites (median cover abundance = 103%) (Fig. 4A), had low cover abundance (7%) in sites of moderate fire exposure, and was close to being absent (0%) from sites of low fire exposure. Lateral spreaders were almost absent (median cover abundance = 0%) in sites subject to high fire exposure, moderately abundant (47%) in sites of moderate fire exposure, and most abundant (103%) in sites subject to low fire exposure. Vertical growers were largely absent from moderate and high fire exposure sites (median cover abundance = 0%) but relatively abundant (38%) in sites subject to low fire exposure.

The canopy cover, and thus canopy overlap, differed significantly between the fire exposure categories (*F* = 11.467; *P* < 0.001); it was significantly higher (mean canopy cover of 145%) in low fire exposure than high (101%) (*P* < 0.001) and moderate (74%) (*P* = 0.014) fire exposure, and significantly higher in high fire exposure than in moderate fire exposure (*P* < 0.001). Dune thicket in low fire exposure sites was significantly taller (median height of 4 m) than in moderate fire-exposed sites (3 m) and shortest in high fire-exposed sites (0.6 m) (*H* = 1,631.494; *P* < 0.001) (Fig. 4B).

## Discussion

### Species diversity and composition

The floristic units we identified in relation to three fire exposure categories corresponded to communities recognized in Cowling’s (1984) phytosociological study undertaken in our study area. Thus, the high fire exposure sites resembled Cowling’s *Restio eleocharis—Maytenus procumbens* dune fynbos-thicket community, characterized by a high cover of hedge-forming thicket canopy species, notably *Maytenus procumbens, Olea exasperata, Euclea racemosa, Lauridia tetragona, Searsia glauca, S. crenata, S. laevigata* and *Rapanea gilliana* (Endangered) (Victor, 2006), as well as a variety of fynbos shrubs and herbs. This community is described as a successional phase of the fynbos-thicket transition in these dune landscapes. The moderately fire-exposed sites correspond to Cowling’s (1984) *Mystroxylon aethiopicum—Cussonia thyrsiflora* dune thicket community. Dominant canopy species include *Mystroxylon aethiopicum, Pterocelastrus tricuspidatus, Sideroxylon inerme, Chionanthus foveolatus, Cassine peragua* and *Scutia myrtina*. Cowling (1984) sampled only one site that resembled our low fire exposure unit. Here the dominant canopy species comprised a mix of forest (*e.g., Canthium spinosum, Zanthoxylon capense*) and thicket (*e.g., Mystroxylon aethiopicum, Sideroxylon inerme*) species. Cowling (1984) did not consider the role of fire in maintaining the boundary between the dune communities of the study area, stressing instead the roles of soil moisture and drainage, whereas fynbos communities occupied drier, excessively drained dune crest and upper slopes whereas forest and thicket occupied swales which enjoyed higher soil moisture conditions owing to lower depth to the water table. Following Cowling’s (1984) floristic analysis, we term the vegetation of high fire exposure, “fynbos-thicket”, that of moderate fire exposure, “thicket”, and that of low fire exposure, “forest-thicket”.

Our results indicate that the diversity and cover abundance of thicket canopy-forming species are strongly influenced by the degree of fire exposure in the landscape. These results are robust as the various ordinations performed, constrained and unconstrained, showed very similar patterns. Sites at the upper and lower margins of fire exposure, namely fynbos-thicket and forest-thicket, had the highest diversity and compositional distinctiveness. The former was dominated by hedge-forming species *e.g., Olea exasperata, Rapanea gilliana*, and *Robsonodendron maritimum*; the latter including a high abundance of vertically growing species, namely *Acokanthera oppositifolia, Allophylus decipiens, Apodytes dimidiata, Cassine peragua*, *Celtis africana, Dovyalis rhamnoides, Gymnosporia nemorosa* and *Scolopia zeyheri*. These vertical growers are restricted to the tiny forest-thicket patches in the study area and are commonly found in coastal forests throughout the CFR (Mucina, Geldenhuys & Rutherford, 2006; Geldenhuys, 1993; Gadow et al., 2016). The prolonged absence of fire in these sites, and associated build-up of soil organic carbon and persistent shade, enables the recruitment, *via* birds, and persistence of forest species here and nowhere else in the dune landscape (Cowling et al., 1997). On the other hand, thicket, which is subject to moderate fire exposure, has a relatively low diversity of canopy species and is overwhelmingly dominated by *Pterocelastrus tricuspidatus*, with *Euclea racemosa, Sideroxylon inerme* and *Searsia glauca* as subdominants. These species produce large canopies with multiple stems, which likely hinders the establishment of shade-intolerant species found in high fire exposure. The moderate fire return interval might hinder forest species’ (*i.e*., vertical growers) competitive ability and they are likely outcompeted post fire by the dominant lateral spreaders and hedge formers that are strong resprouters (Strydom et al., 2021), thereby limiting species diversity. The higher level of disturbance in fynbos-thicket could potentially be driving diversity as geoxyles and dune-endemic hedge formers are abundant, while the short fire-return intervals keep the lateral-spreading species smaller and shorter, thereby limiting competition for light.

Our results on compositional differences along a fire exposure gradient are broadly consistent with those from other systems where fire-sensitive and fire-dependent systems coexist in the same landscape. The degree of fire exposure is an important influence in the composition of vegetation in forest-fynbos systems in the CFR (Manders, 1990; Geldenhuys, 1994) and in the tropical forest-savanna systems of Africa, Brazil and Australia (Charles-Dominique et al., 2015; Hoffmann et al., 2009; Murphy & Bowman, 2012; Flake et al., 2021a; Flake et al., 2021b; Becket et al., 2022). In both systems, regular fires maintain the more open, fire-dependent ecosystems (fynbos, savanna) by preventing invasion of fire-sensitive forest species (Geldenhuys, 1994; Hoffmann et al., 2009). Our system differs from the aforementioned in that it includes a closed community (thicket) that burns only in unusually fierce fires. Survival of dune thicket species after fire is high (84%) (Strydom et al., 2020) in comparison to that of Cape Afrotemperate forest (45%) (Giddey, Baard & Kraaij, 2022b). This is likely due to differences in fire resiliency traits such as allocation to bark thickness and fire-protected bud banks (Clarke et al., 2013; Charles-Dominique et al., 2015; Corrêa Scalon et al., 2020). While bark thickness of our thicket species has not been investigated, all are capable of resprouting from epicormic, basal and underground bud banks (Strydom et al., 2020; Strydom et al., 2021). Furthermore, thicket species are generally more flammable than forest trees (Calitz, Potts & Cowling, 2015), thus requiring strategies that confer fire resilience traits described above. Consistent with this is the higher allocation of resources among thicket species to vegetative reproduction *via* ramets, than in vertically growing forest trees that allocate more to sexual reproduction, as suggested by the higher numbers of seedlings observed for the latter (Midgley & Cowling, 1993; Midgley, 1996; Kruger, Midgley & Cowling, 1997). This would explain why forest is invariably restricted to fire-free refugia (Geldenhuys, 1994; Govender, Trollope & Van Wilgen, 2006; Cowling & Potts, 2015), whereas the more fire-resilient thicket can persist in fire-exposed sites, and expand into adjacent, more open fynbos-thicket communities in the prolonged absence of fire (Cowling & Hoffman, 2021). Thicket can establish on the drier dune crest and steep upper slopes of fynbos sites but has restricted growth and canopy cover and does not outcompete fynbos on these sites (Cowling & Hoffman, 2021), possibly because seedlings and ramets are outcompeted for soil moisture by the mass of fine roots associated with fynbos shrubs (Lu et al., 2022).

### Structural composition

The degree of fire exposure in our dune landscape profoundly influences the structural composition of vegetation as represented by the architecture of dune thicket canopy species. This is also true of other systems such as forest-savanna (Higgins et al., 2007; Smit et al., 2010; Ryan, 2002) and forest-fynbos mosaics (Kruger & Bigalke, 1984; van Wilgen, Higgins & Bellstedt, 1990). At high fire exposure, thicket species comprise a diverse array of hedge formers characterized by many relatively thin and low shoots arising from robust underground stems (Cowling, 1983; Grobler & Cowling, 2021; Fig. S8). This architecture is consistent with the geoxylic structure of species commonly found in the fire-swept African savannas (the “underground forests” of White, 1976; Meerts, 2017), where it is regarded as an adaptation to frequent fire (Maurin et al., 2014; Lamont, He & Pausas, 2017; Pausas et al., 2018). Interestingly, many of the geoxylic hedge formers in fynbos-thicket are endemic to the dune landscapes of the CFR, namely *Olea exasperata, Rapanea gilliana, Robsonodendron maritimum* and *Searsia crenata* (Cowling, 1984; Grobler & Cowling, 2021). This growth form can provide an alternative means of surviving fire, allowing for multiple stems to occupy an extensive area and protecting woody biomass and buds belowground (Maurin et al., 2014; Wigley et al., 2019). Indeed, hedge formers produce numerous ramets after fire, similar in abundance to seedlings (genets) produced by non-sprouting fynbos shrubs in fynbos-thicket (Cowling & Pierce, 1988, Fig. S9). Geoxylic hedge formers require frequent fire for persistence, otherwise they are outcompeted by taller shrubs and trees that invest more in above ground biomass (Fidelis et al., 2014). This is evident in moderate and low fire-exposed dune thicket where the geoxylic species are largely absent.

Under moderate fire exposure, hedge formers are largely replaced by lateral spreaders – notably *Pterocelastrus tricuspidatus* (Fig. S10) – which invest more in aboveground shoots but still retain fire resilience. The thicket community unit associated with this fire environment comprises a closed, multi-stemmed, 3–6 m high vegetation. Vertical growing species are represented by seedlings (Cowling et al., 1997) and occasional saplings which are in the center of clumps as understorey or emergent individuals.

Under conditions of low fire exposure, thicket is invaded by vertical-growing forest tree species like *Apodytes dimidiata*, *Chionanthus foveolatus*, *Celtis africana, Scolopia zeyheri*, and *Psydrax obovata*, which establish from bird-dispersed propagules (Cowling et al., 1997). We propose that vertical growers will ultimately dominate thicket only in those sites that are protected from regular fire, as is the case for Afrotemperate forest trees in fynbos-dominated landscapes (Geldenhuys, 1994; Calitz, Potts & Cowling, 2015). The lower flammability of forest species (Calitz, Potts & Cowling, 2015), as well as soil amelioration *via* nutrient input from the prolonged deposition of litter (Cowling, 1984), would further promote forest development. Similar processes are associated with the invasion of fynbos by Afrotemperate forest (Coetzee, Bond & Wigley, 2015; Cowling & Potts, 2015) and the invasion of tropical African savanna by rainforest (Hoffmann, Orthen & Franco, 2004; Becket et al., 2022; Flake et al., 2021a; Flake et al., 2021b; Geiger et al., 2011).

In dune thicket, certain species that predominantly present as lateral spreaders, *i.e., Sideroxylon inerme, Mystroxylon aethiopicum* and *Pterocelastrus tricuspidatus*, have high phenotypic plasticity (Fig. S11) (Strydom et al., 2021) which enables persistence in low, moderate and high fire exposure environments. Under low fire exposure these species are dominant and can shed lateral branches in favor of fewer vertical branches, shifting from a lateral spreader to a vertical grower *via* architectural modification (Halle, Oldeman & Tomlinson, 1978; Strydom et al., 2021). In high fire exposure conditions, they can adopt hedge-like architectures, where *Sideroxylon inerme* has a fair canopy dominance. Other species, such as *Euclea racemosa* and *Olea exasperata*, mostly dominant in high fire exposure sites as hedge formers, can – because of competition from taller-growing species – invest resources into one or more stems that reach canopy height, thereby adopting a vertical grower architecture. Similarly, the architecture of highly plastic *Vachellia karroo* (Hayne) Banfi & Galasso is determined by its environment; in light-limited forest, individuals resemble vertical growers, in fire-swept open savanna they resemble lateral growers, and in fire-free arid shrubland, they resemble hedge formers (Archibald & Bond, 2003). Interestingly, we have observed no case of a predominantly vertical-growing species being sufficiently plastic to adopt lateral-spreading or hedge-forming architectures. This likely underpins their inability to persist in landscapes subject to moderate and high fire exposure.

In high fire exposure the underground stem (geoxylic) structure of certain hedge formers (*Euclea racemosa, Olea exasperata, Rapanea gilliana, Searsia laevigata*) is likely an adaptation to recurrent fire (Maurin et al., 2014; Lamont, He & Pausas, 2017; Meller et al., 2022) and, given our knowledge of the sequential temporal emergence of dominant disturbance regimes (herbivory *vs*. fire) through evolutionary history (Cowling et al., 2005; Keeley & Rundel, 2005; Pennington et al., 2010), we would expect these geoxylic species to be more recently diversified than their laterally-spreading or vertically-growing sister species. Examples of closely related pairs of species from the Cape that have different architectures include: *Rapanea gilliana*, a hedge-forming geoxylic shrub endemic to fire-exposed dune fynbos-thicket in the southeastern Cape, which is likely derived from the vertical-growing, arborescent *Rapanea melanophloeos*, a typical component of local forests (Cowling, 1983); and *Olea exasperata*, another geoxylic hedge-former restricted to CFR coastal dunes, which is derived from the vertical-growing forest species *Olea capensis* (Besnard et al., 2009). Based on the evidence presented above, we hypothesise that the vertical growing and lateral spreading architectures would be associated with basal lineages, whereas hedge formers, especially geoxylic species, are likely associated with lineages of younger age. However, comprehensive phylogenetic data are lacking to test this hypothesis.

## Conclusions

The coastal dunes of the southeastern CFR comprise three biomes – forest, thicket and fynbos – that are associated with increasing exposure to recurrent fire. We show here that fire exposure has a profound effect on the floristic and structural composition of these biomes. Forest-thicket, which seldom or ever burns, comprises a diverse assemblage of laterally spreading shrubs and vertically-growing trees, the latter being restricted to small populations in the rare fire-free habitats of the dune landscape. Many of the vertical growers are poor resprouters that recruit mainly from seedlings. Fynbos-thicket is similarly diverse, comprising many hedge-forming species, most of which have extensive belowground stems indicative of a geoxylic structure. These species are capable of vigorous post-fire resprouting and include several dune-endemic taxa that do not grow in the other biomes. Thicket, which is subject to intermediate fire exposure comprises a relatively depauperate flora of laterally-spreading, multi-stemmed and fire-resilient shrubs, some of which show high architectural plasticity (*i.e.*, they can grow as either hedge formers or vertical foragers, depending on the degree of fire exposure). We suggest that the primary selective force for sprouting in these thicket lineages was browsing by megaherbivores in the Paleogene; fire likely became a factor of the evolution of subtropical grass-dominated ecosystems during the Neogene. However, more research is required to test this hypothesis. More research is also required on the belowground architecture of these thicket lineages and how this influences competitive interactions between architectural types in relation to fire exposure.

## Supplemental Information

10.7717/peerj.14310/supp-1 Supplemental Information 1 Reverse chronical series of aerial images available for the greater St Francis area in the southeastern Cape, South Africa. The 2016 aerial image shows the outline of the 2016 fire as well as the extent of a photo taken by drone of the study area depicting the fire exposure categories.

10.7717/peerj.14310/supp-2 Supplemental Information 2 Rank abundance curve of nine dune thicket species across three fire exposure categories.

10.7717/peerj.14310/supp-3 Supplemental Information 3 Maximal distance-based redundancy analysis (dbRDA) ordination plot and for cover abundance data of dune thicket species. The maximal distance-based Redundancy Analysis (dbRDA) ordination was plotted using Bray-Curtis dissimilarity matrix based on cover abundance data of species and projected onto two-dimensional space. The shapes (square, circle, triangle) are transects colored according to their location in pre-determined fire exposure categories for dune thicket (green = low fire exposure; blue = moderate fire exposure; orange = high fire exposure). The dashed ellipses indicate 95% confidence intervals around the centroids of each of these categories. The effect of aspect has not been controlled for in this dbRDA. The legend (color coded) shows the architectural guild which is most common in the respective fire exposure categories.

10.7717/peerj.14310/supp-4 Supplemental Information 4 Cluster analysis (CA) with the use of the Bray-Curtis dissimilarity matrix (Ward’s method) based on cover abundance data of species across 51 transects. Low 1–17 = low fire exposure transects; Mod 1–17 = moderate fire exposure transects; High 1–17 = high fire exposure transects. The dashed line indicates where the dendrogram was cut (linkage distance = 3). The rectangles delineate the three fire exposure categories.

10.7717/peerj.14310/supp-5 Supplemental Information 5 Non-Metric Multidimensional Scaling (NMDS) ordination plot for cover abundance of dune thicket species. The ordination was plotted using the Bray-Curtis dissimilarity matrix based on cover abundance data of species and projected onto two-dimensional space. (a) The shapes (square, circle, triangle) are transects colored according to their location in pre-determined fire exposure categories for dune thicket (green = low fire exposure; blue = moderate fire exposure; orange = high fire exposure) and (b) species according to their location in the fire exposure categories. The dashed ellipses indicate 95% confidence intervals for these categories. NMDS solution: k = 2, stress = 16.1%, non-metric R^2^= 0.974, linear R^2^= 0.874. NMDS axis 1 reflects the fire exposure gradient, which is aligned along the axis of most variation in species cover abundance. The legend (color coded) shows the architectural guild which is most common in the respective fire exposure categories.

10.7717/peerj.14310/supp-6 Supplemental Information 6 Cluster analysis (CA) with the use of the Bray-Curtis dissimilarity matrix (Ward’s method) based cover abundance data of architectural guilds across 51 transects. Low 1–17 = low fire exposure transects; Mod 1–17 = moderate fire- exposure transects; High 1–17 = high fire exposure transects. The dashed line indicates where the dendrogram was cut (linkage distance = 5). The rectangles delineate the three architectural guild cover abundance between three fire exposure categories.

10.7717/peerj.14310/supp-7 Supplemental Information 7 Non-Metric Multidimensional Scaling (NMDS) ordination plot for cover abundance of dune thicket architectural guilds. The ordination was plotted with the use of the Bray-Curtis dissimilarity matrix based on cover abundance data of architectural guilds and projected onto two-dimensional space. The shapes (triangle, square and circle) are transects colored according to their location in predetermined fire exposure categories (green = low fire exposure; blue = moderate fire exposure; orange = high fire exposure) and the cross dashed ellipses indicate 95% confidence intervals for these categories. Because of extreme structural homogeneity in high fire exposure, 14 of 17 transects plotted on top each other (indicated by an asterisk in the triangle). NMDS solution: k = 2, stress = 1.4%, linear R2 = 0.999, non-metric R2 = 1. NMDS axis 1 reflects the fire exposure gradient, which is aligned along the axis of most variation in architectural guild cover abundance. The legend (color coded) shows the architectural guild which is most common in the respective fire exposure categories.

10.7717/peerj.14310/supp-8 Supplemental Information 8 Geoxylic habit of *Euclea racemosa* (dark brown below-ground stems).

10.7717/peerj.14310/supp-9 Supplemental Information 9 *Searsia laevigata* ramets recruiting from belowground stems 2 months post-fire in a coastal dune setting exposed to high fire frequency. Cape St Francis.

10.7717/peerj.14310/supp-10 Supplemental Information 10 The lateral spreader *Pterocelastrus tricuspidatus* (background) emerging from a long unburnt fynbos-thicket, dominated by the hedge-forming *Olea exasperata* (foreground). Cape St Francis.

10.7717/peerj.14310/supp-11 Supplemental Information 11 Phenotypic plasticity displayed by a single species (*Sideroxylon inerme*), adopting all of the architectural guilds: (a) hedge former, (b) lateral spreader, and (c), vertical grower.

10.7717/peerj.14310/supp-12 Supplemental Information 12 Summary of anecdotal accounts and aerial imagery consulted to establish a history of fire occurrence in and around Cape St Francis.

10.7717/peerj.14310/supp-13 Supplemental Information 13 The slope, aspect and soil type of if the transects located in the different fire exposure categories (L1-17, low fire exposure; M1-17, moderate fire exposure; H1-17, high fire exposure).

10.7717/peerj.14310/supp-14 Supplemental Information 14 ADONIS analysis for cover abundance of dune thicket species across three fire exposure categories. Permutational Multivariate Analysis of Variance shows that there is a significant difference between the three fire exposure categories (F = 15.051, *P* = 0.001). Pairwise multilevel comparison shows that all fire exposure categories are significantly different from each other (adjusted *P* = 0.003 for all comparisons).

10.7717/peerj.14310/supp-15 Supplemental Information 15 ADONIS analysis for cover abundance of three architectural guilds across three fire exposure categories. Permutational Multivariate Analysis of Variance shows that there is a significant difference between the three fire exposure categories (F = 59.237, *P* = 0.001). Pairwise multilevel comparison shows that all fire exposure categories are significantly different from each other (adjusted *P* = 0.003 for all comparisons).

10.7717/peerj.14310/supp-16 Supplemental Information 16 Raw data for subtropical dune thicket canopy species in the dune landscape of Cape St Francis. The species architectural traits, cover abundance and fire exposure categories.

## Additional Information and Declarations

### Data Availability

The following information was supplied regarding data availability: The raw data is available in the Supplemental File. The R code (db-RDA, CA and NMDS statistical analysis and ordinations) is available at Zenodo. https://doi.org/10.5281/zenodo.7192748.
