# Supplementary material for: Canopy plant composition and structure of Cape subtropical dune thicket are predicted by the levels of fire exposure
Source: PeerJ. 2022 Nov 8;10:e14310. doi: 10.7717/peerj.14310 (PMC9651048; doi:10.7717/peerj.14310)

**Supplemental Figure 1:** Reverse chronical series of arial images available for the greater St Francis area in the southeastern Cape, South Africa. The 2016 aerial image shows the outline of the 2016 fire as well as the extent of a photo taken by drone of the study area depicting the fire-exposure categories.


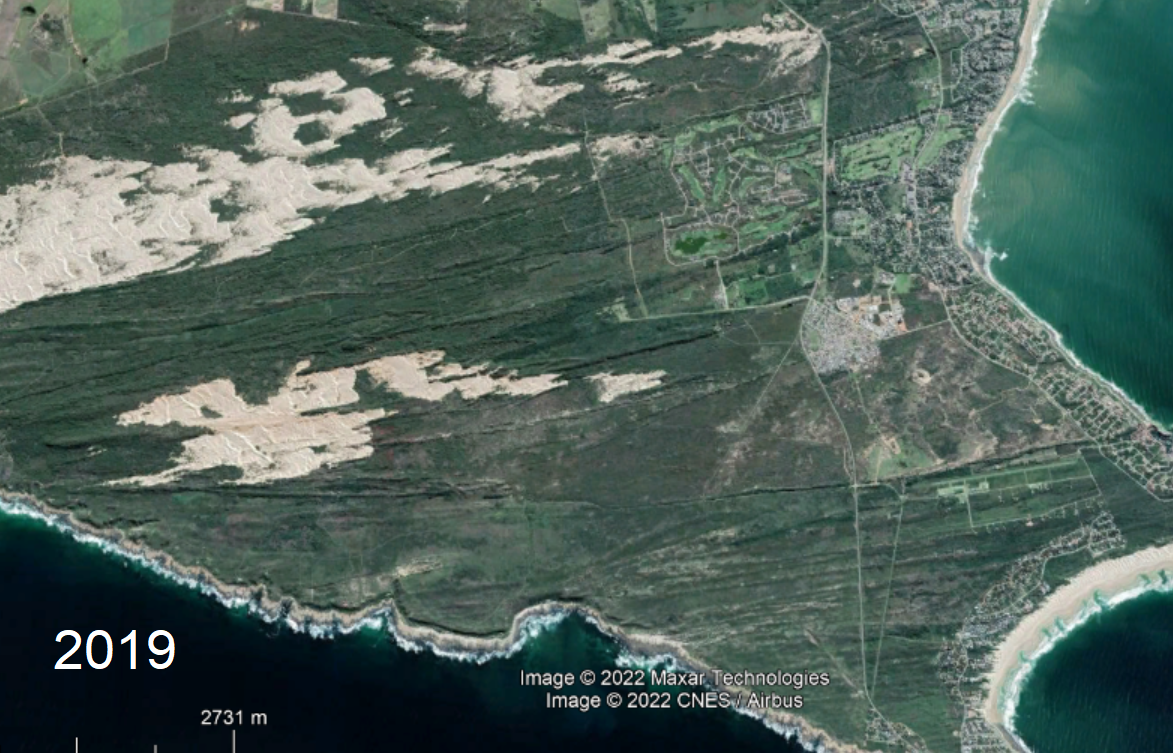


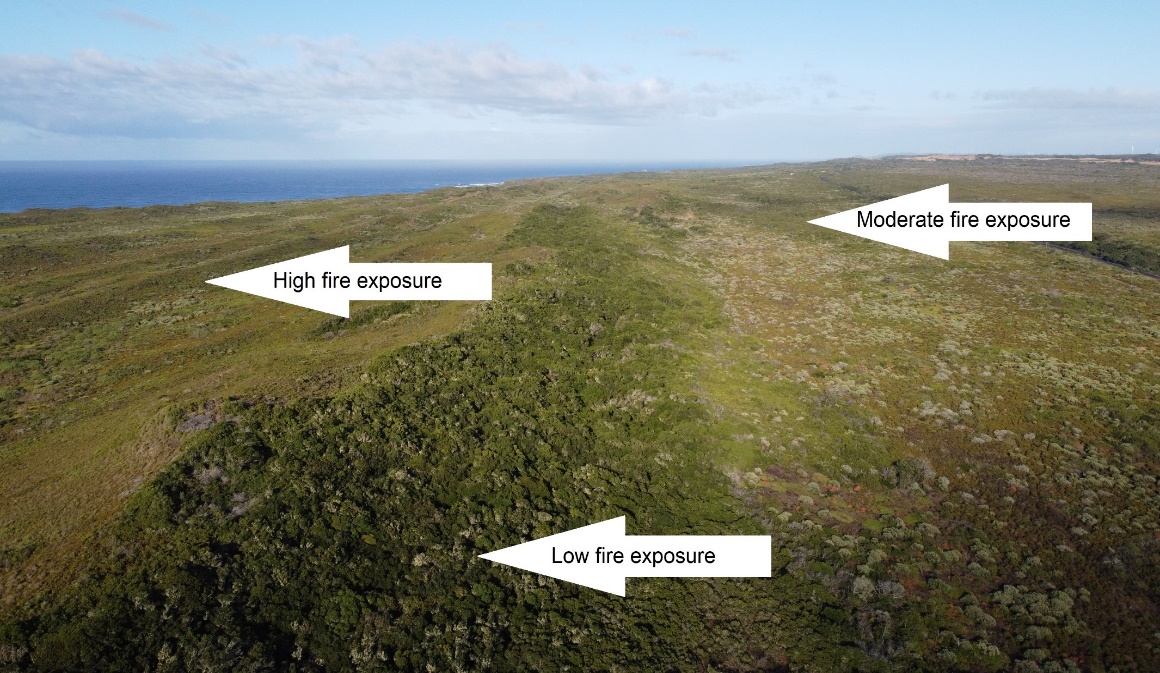

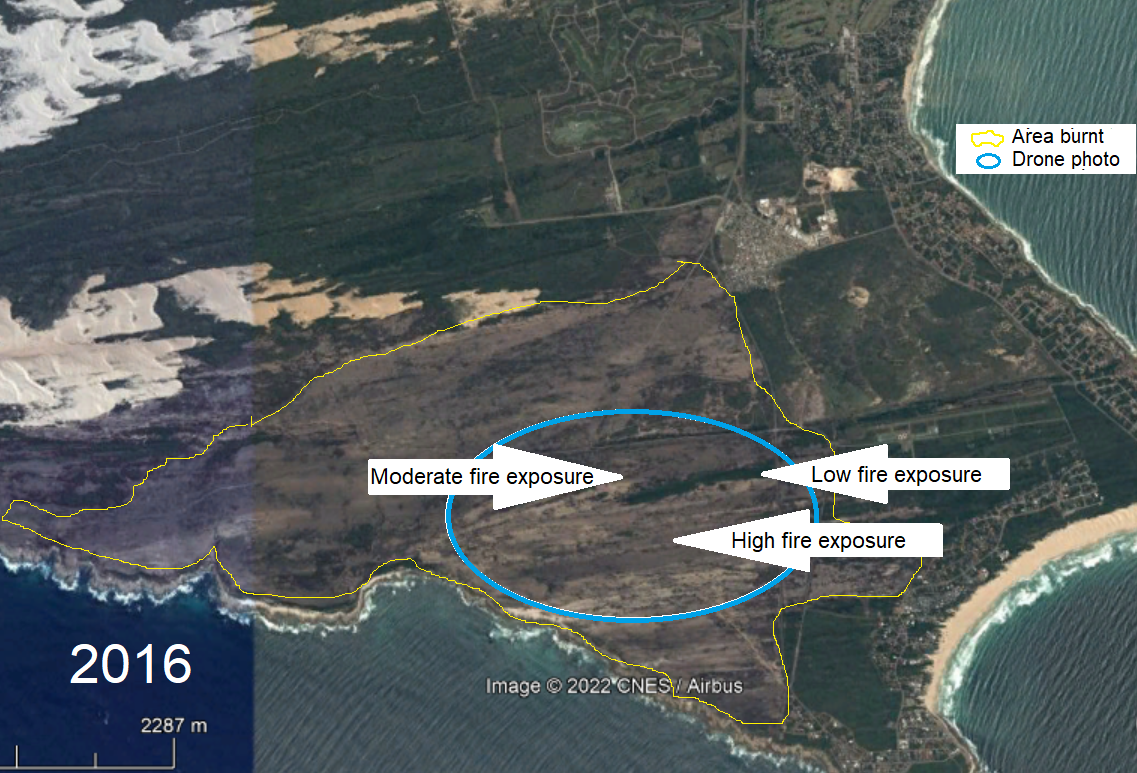


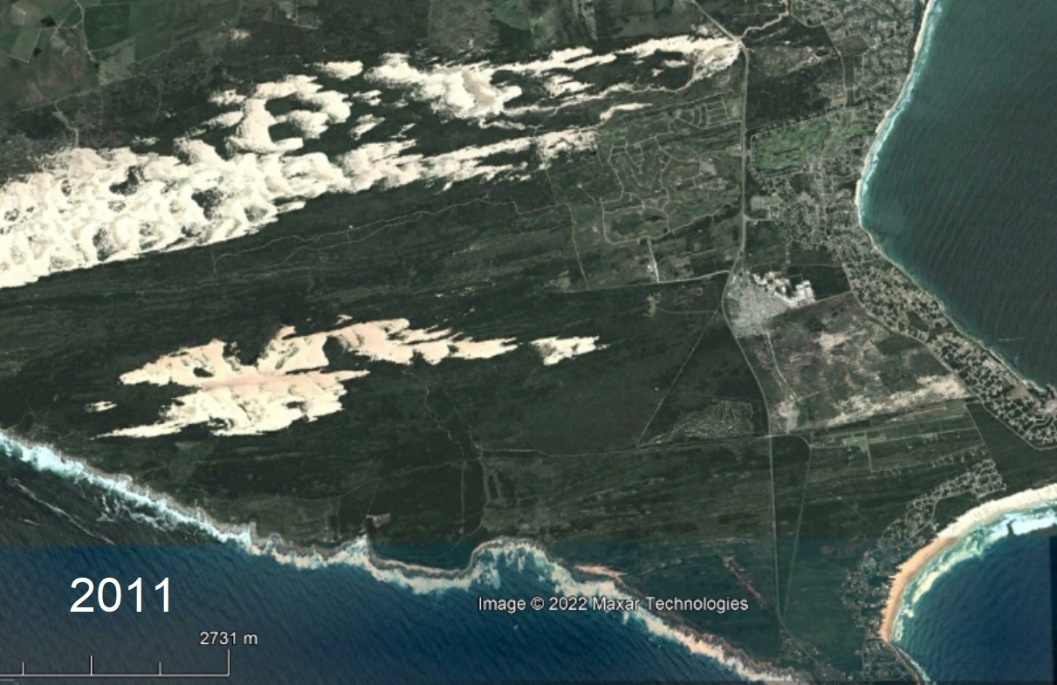

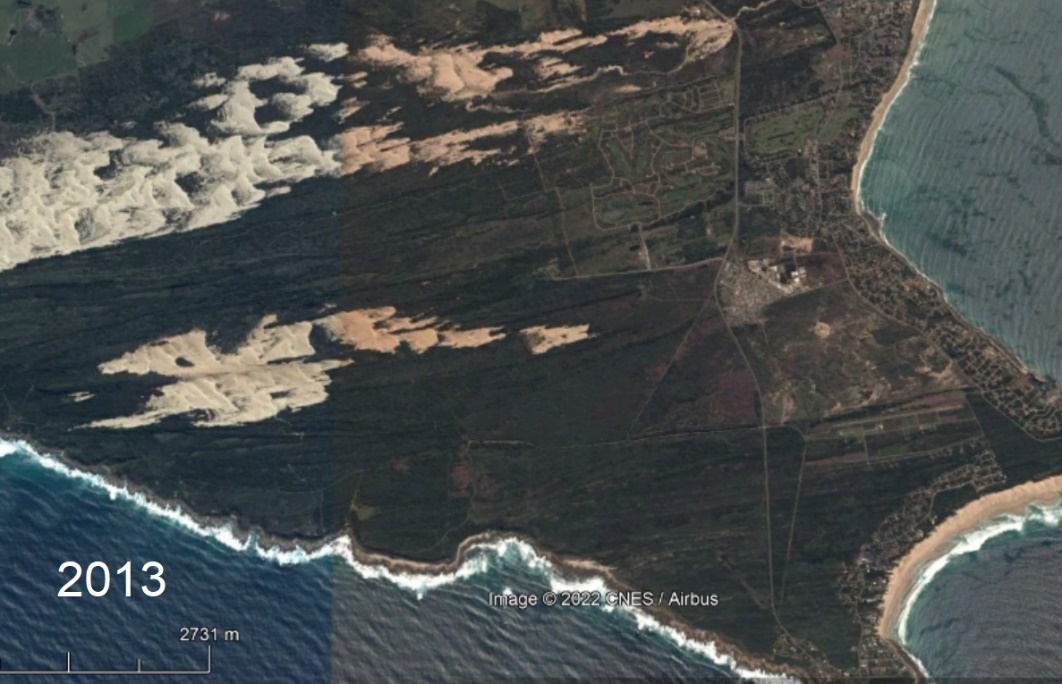

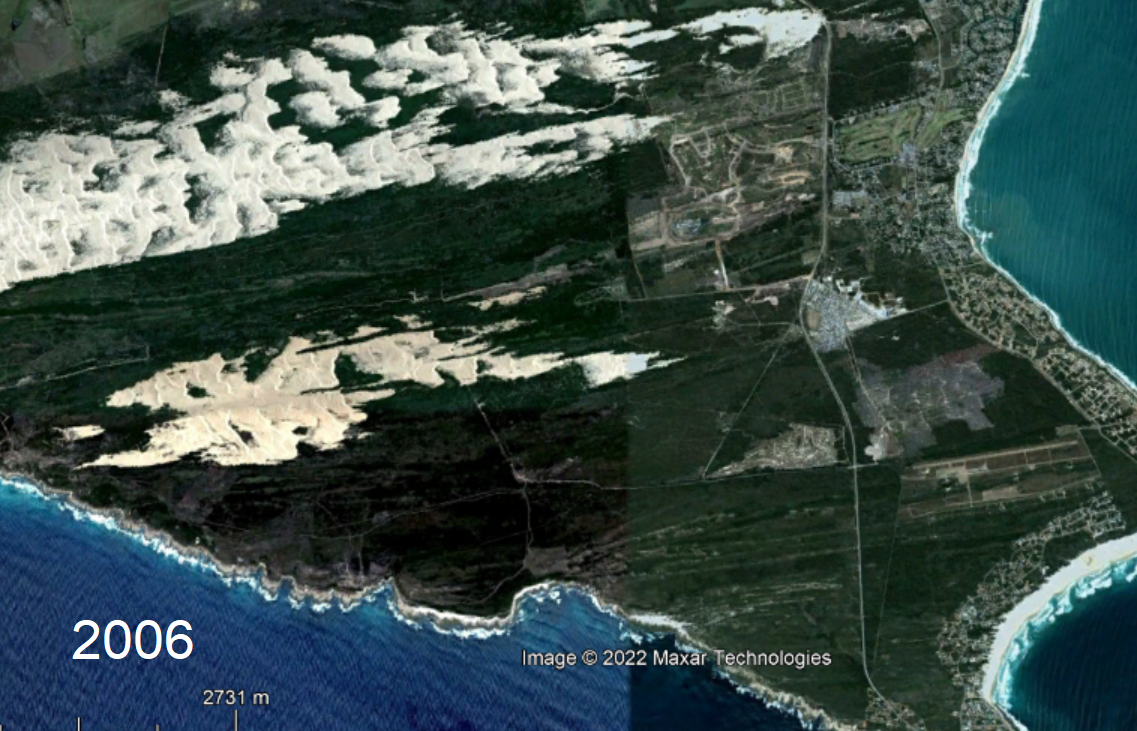

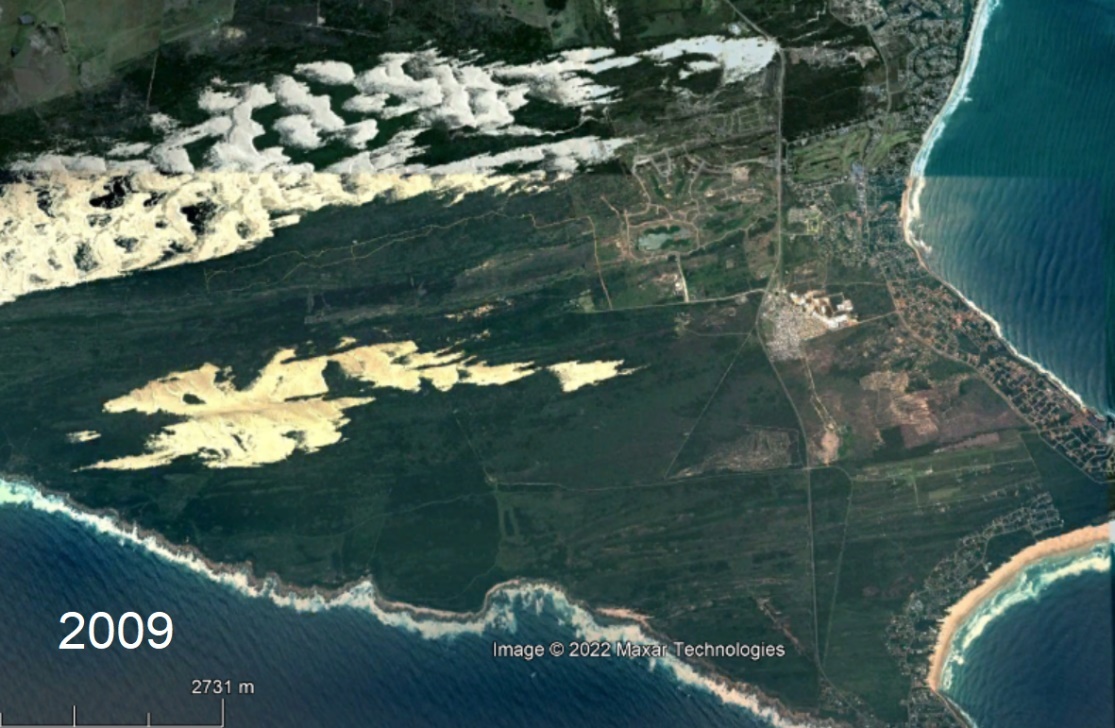

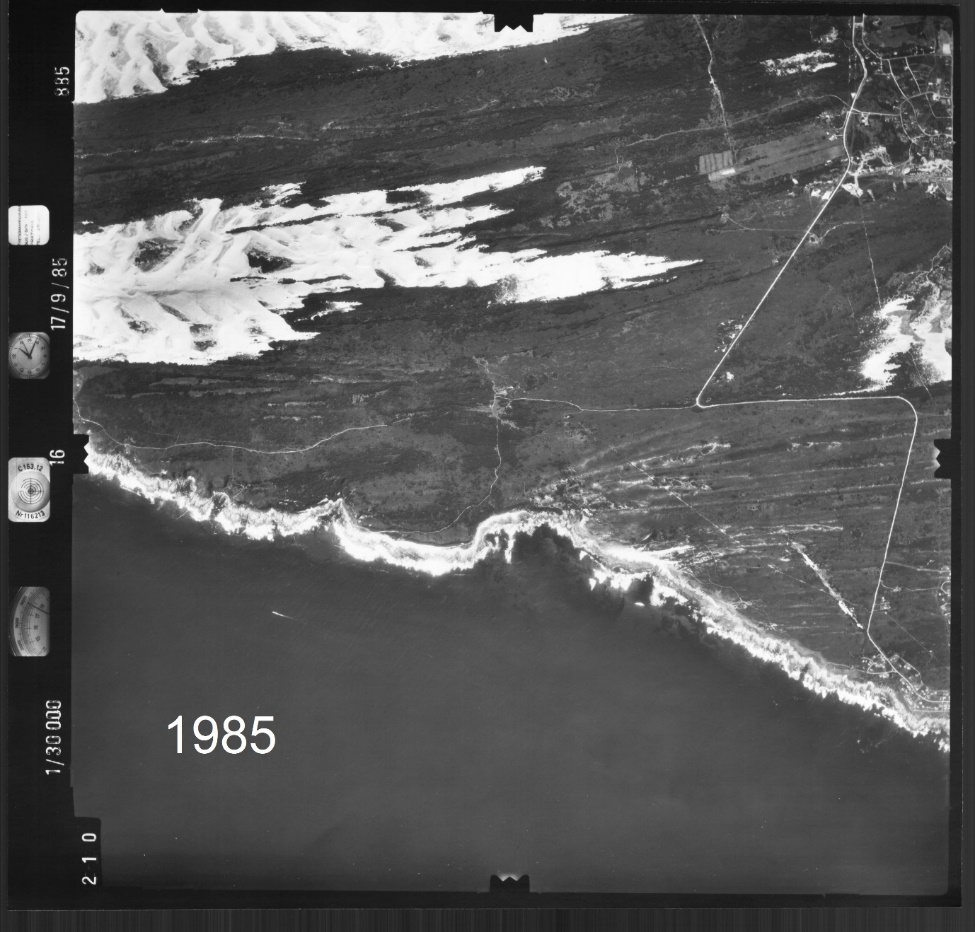

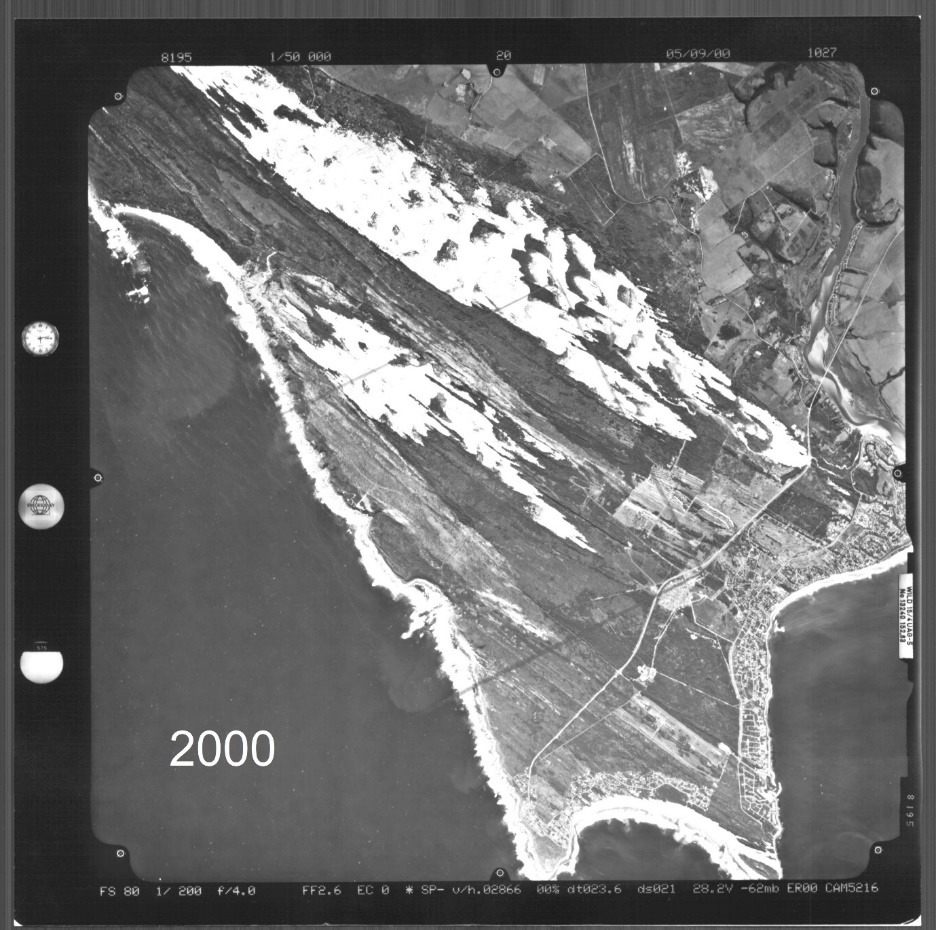

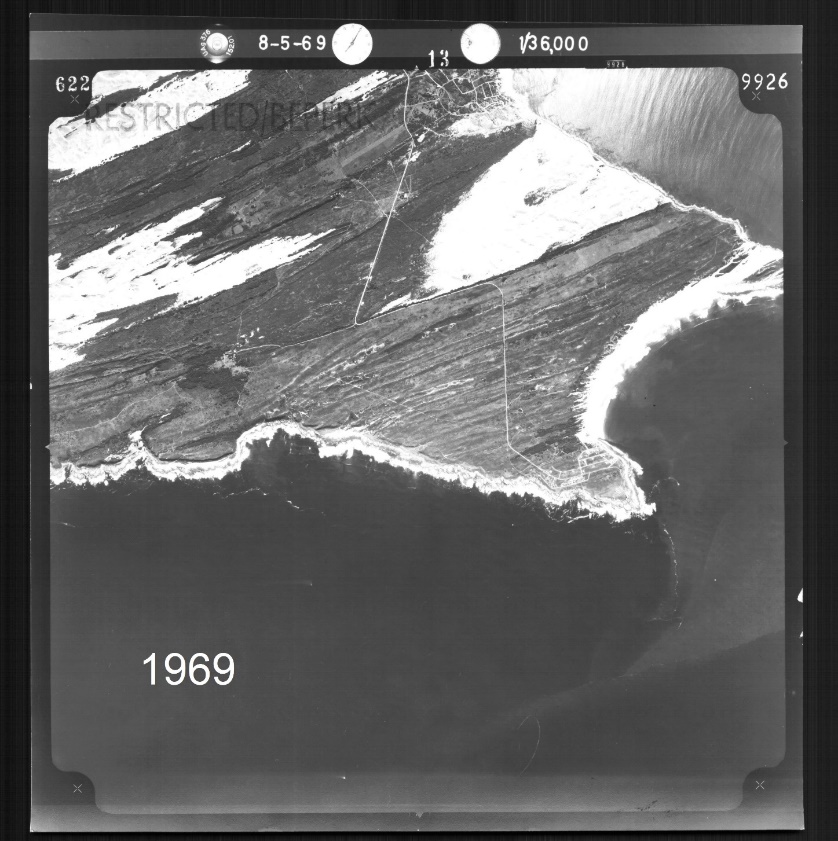

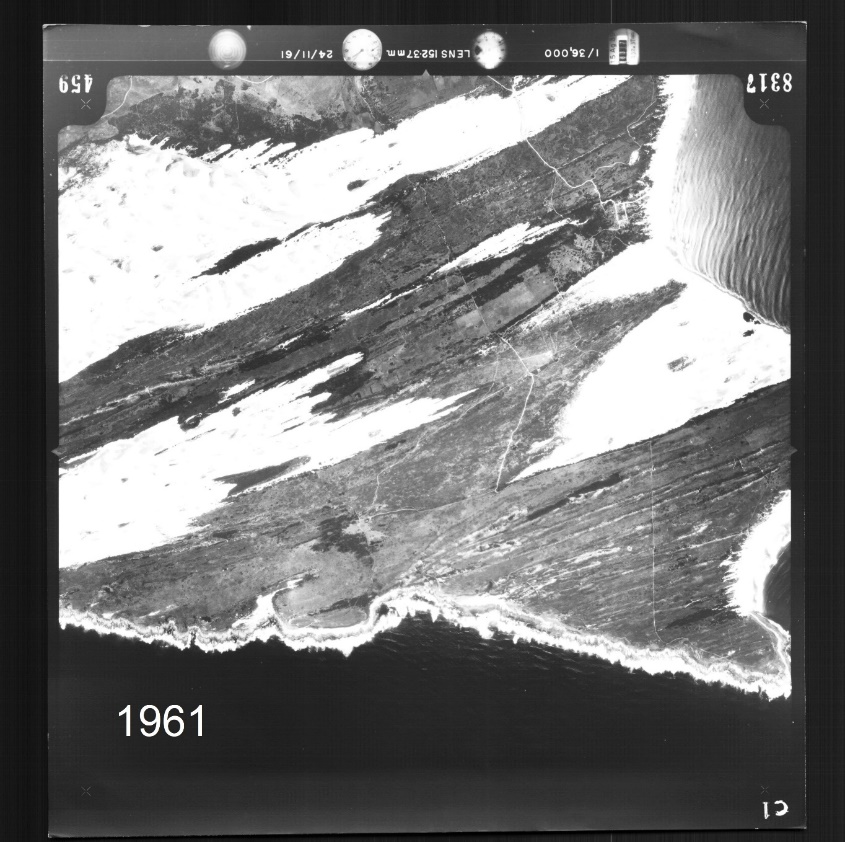

Supplement: Supplemental Information 1 — The 2016 aerial image shows the outline of the 2016 fire as well as the extent of a photo taken by drone of the study area depicting the fire exposure categories. [file peerj-10-14310-s001.docx]
