# Supplementary figures and images for: Canopy plant composition and structure of Cape subtropical dune thicket are predicted by the levels of fire exposure
Source: PeerJ. 2022 Nov 8;10:e14310. doi: 10.7717/peerj.14310 (PMC9651048; doi:10.7717/peerj.14310)

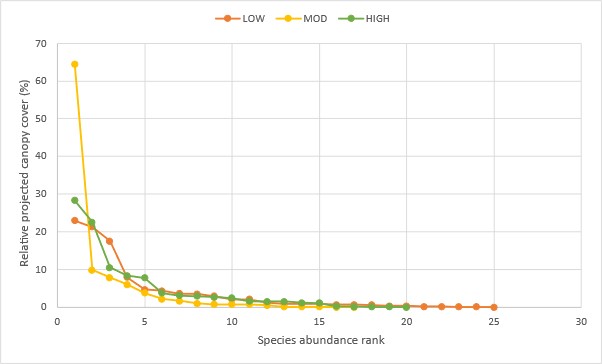

Supplement: Supplemental Information 2 [file peerj-10-14310-s002.jpg]

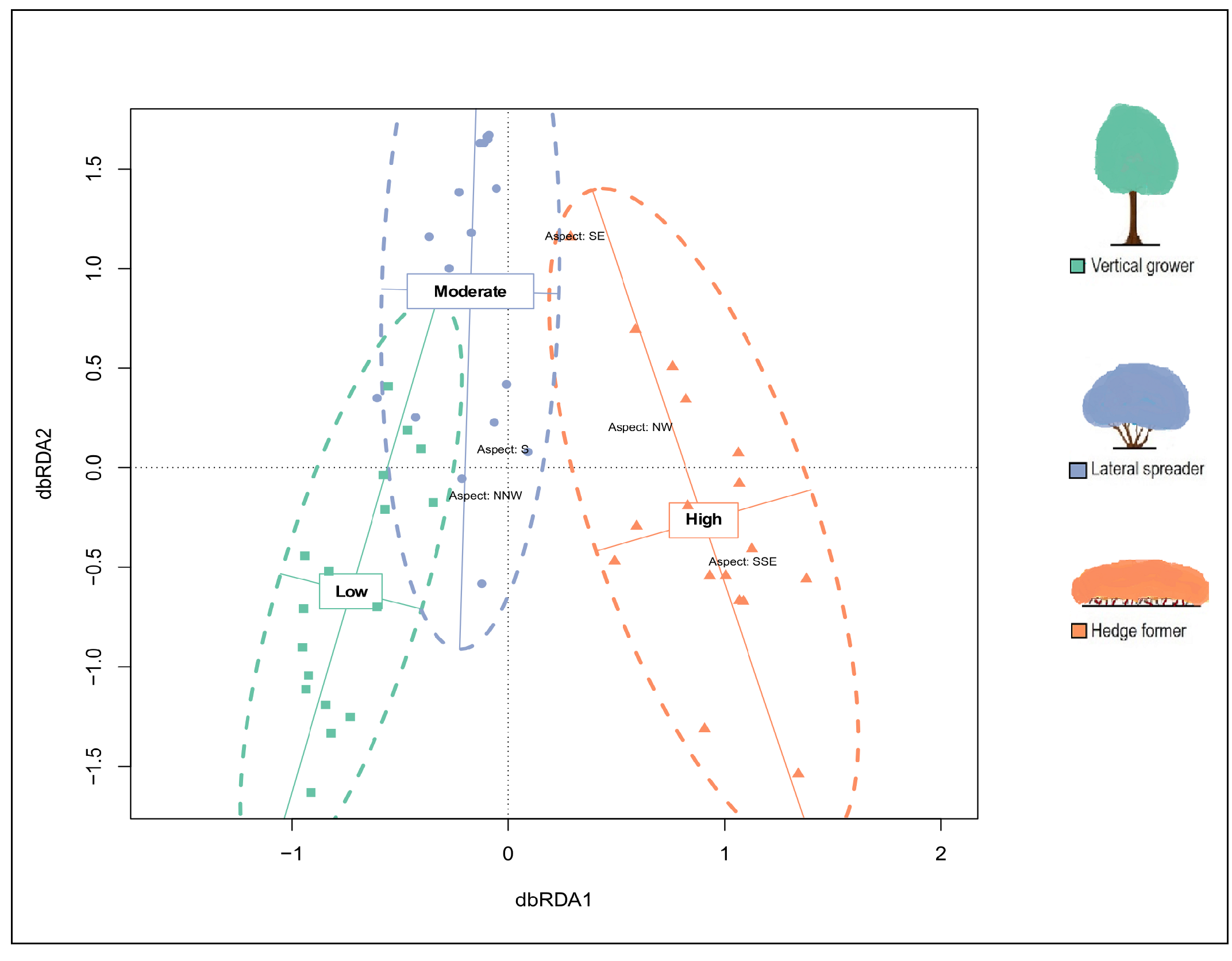

Supplement: Supplemental Information 3 — The maximal distance-based Redundancy Analysis (dbRDA) ordination was plotted using Bray-Curtis dissimilarity matrix based on cover abundance data of species and projected onto two-dimensional space. The shapes (square, circle, triangle) are transects colored according to their location in pre-determined fire exposure categories for dune thicket (green = low fire exposure; blue = moderate fire exposure; orange = high fire exposure). The dashed ellipses indicate 95% confidence intervals around the centroids of each of these categories. The effect of aspect has not been controlled for in this dbRDA. The legend (color coded) shows the architectural guild which is most common in the respective fire exposure categories. [file peerj-10-14310-s003.png]

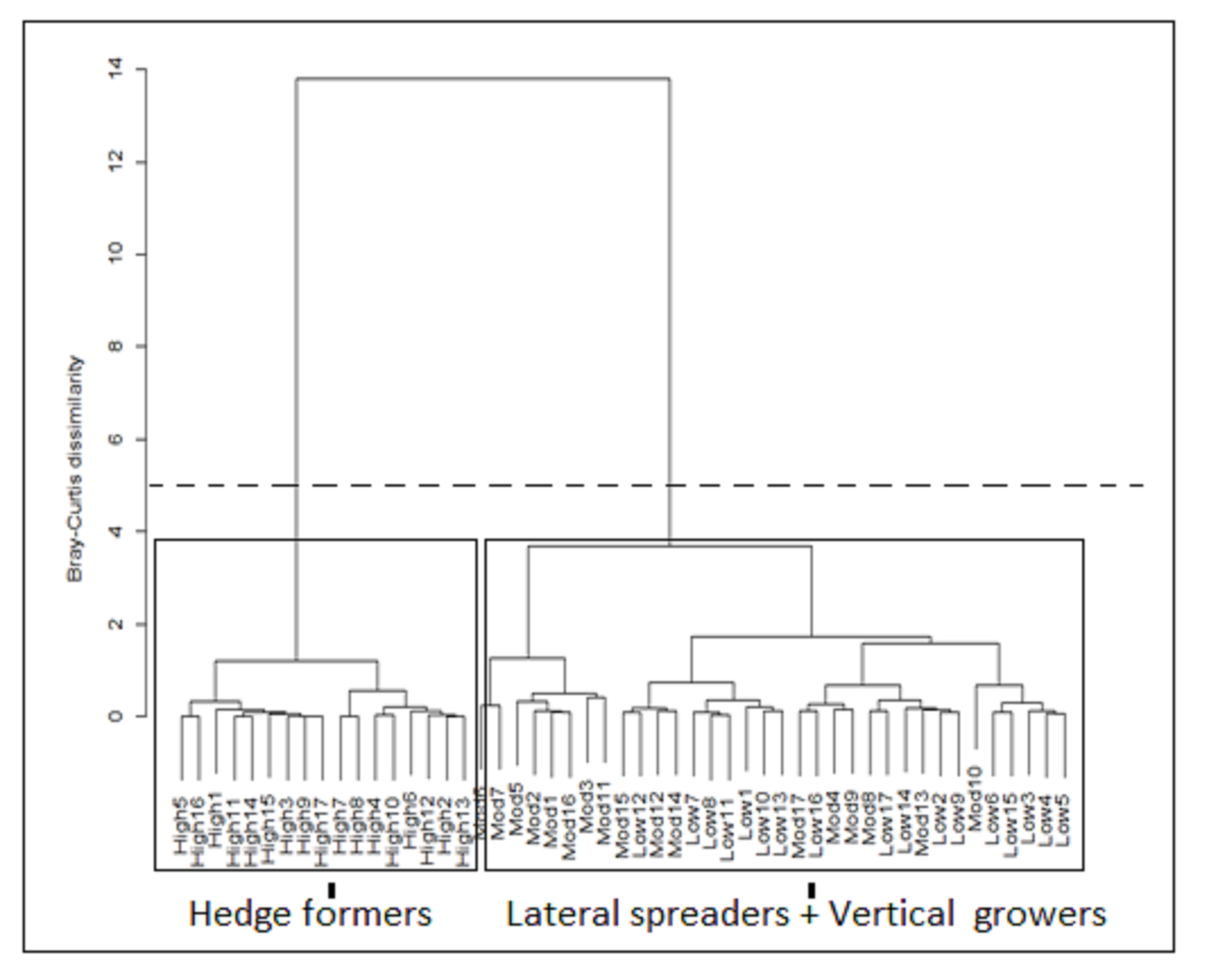

Supplement: Supplemental Information 4 — Low 1–17 = low fire exposure transects; Mod 1–17 = moderate fire exposure transects; High 1–17 = high fire exposure transects. The dashed line indicates where the dendrogram was cut (linkage distance = 3). The rectangles delineate the three fire exposure categories. [file peerj-10-14310-s004.png]

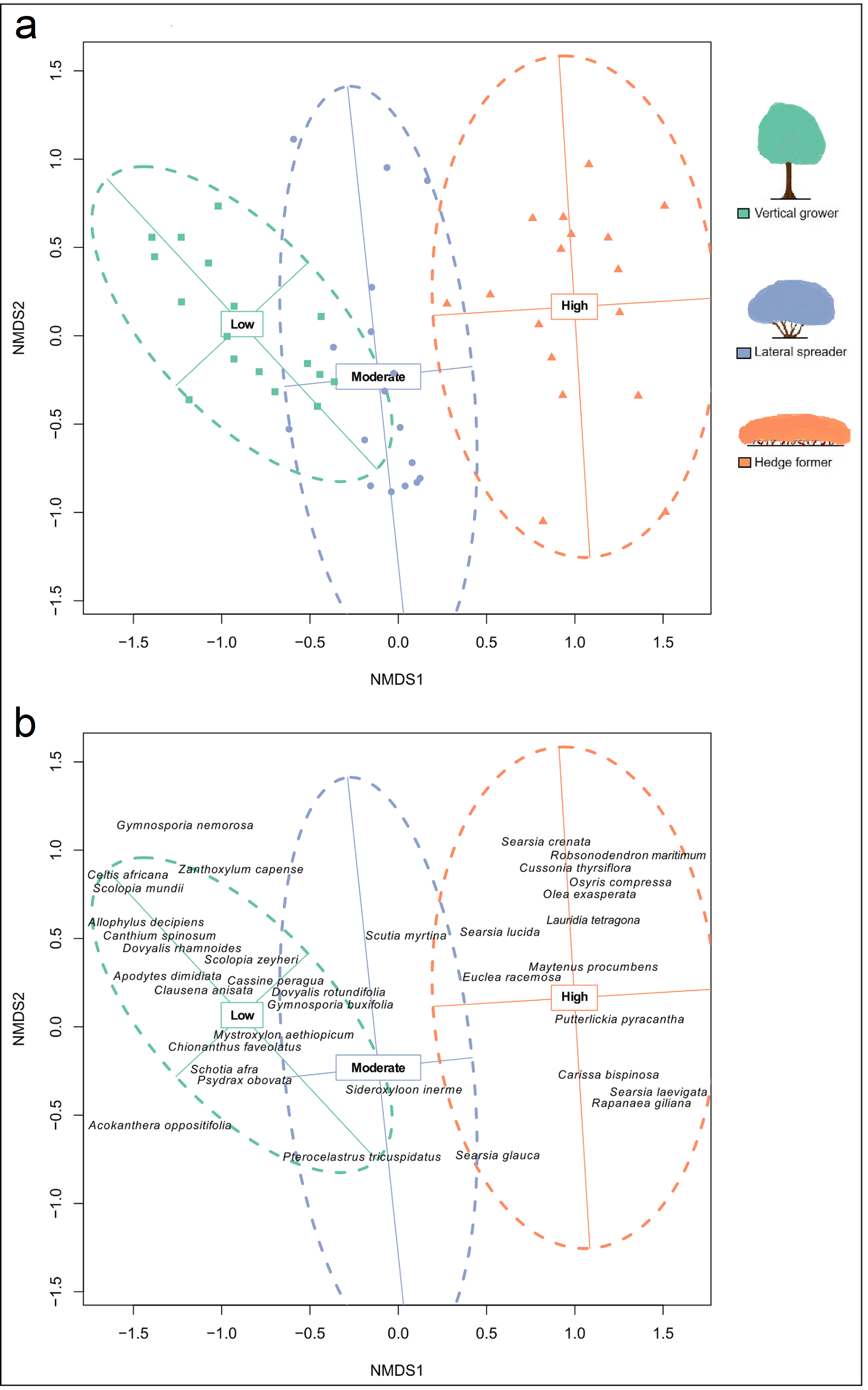

Supplement: Supplemental Information 5 — The ordination was plotted using the Bray-Curtis dissimilarity matrix based on cover abundance data of species and projected onto two-dimensional space. (a) The shapes (square, circle, triangle) are transects colored according to their location in pre-determined fire exposure categories for dune thicket (green = low fire exposure; blue = moderate fire exposure; orange = high fire exposure) and (b) species according to their location in the fire exposure categories. The dashed ellipses indicate 95% confidence intervals for these categories. NMDS solution: k = 2, stress = 16.1%, non-metric R2= 0.974, linear R2= 0.874. NMDS axis 1 reflects the fire exposure gradient, which is aligned along the axis of most variation in species cover abundance. The legend (color coded) shows the architectural guild which is most common in the respective fire exposure categories. [file peerj-10-14310-s005.png]

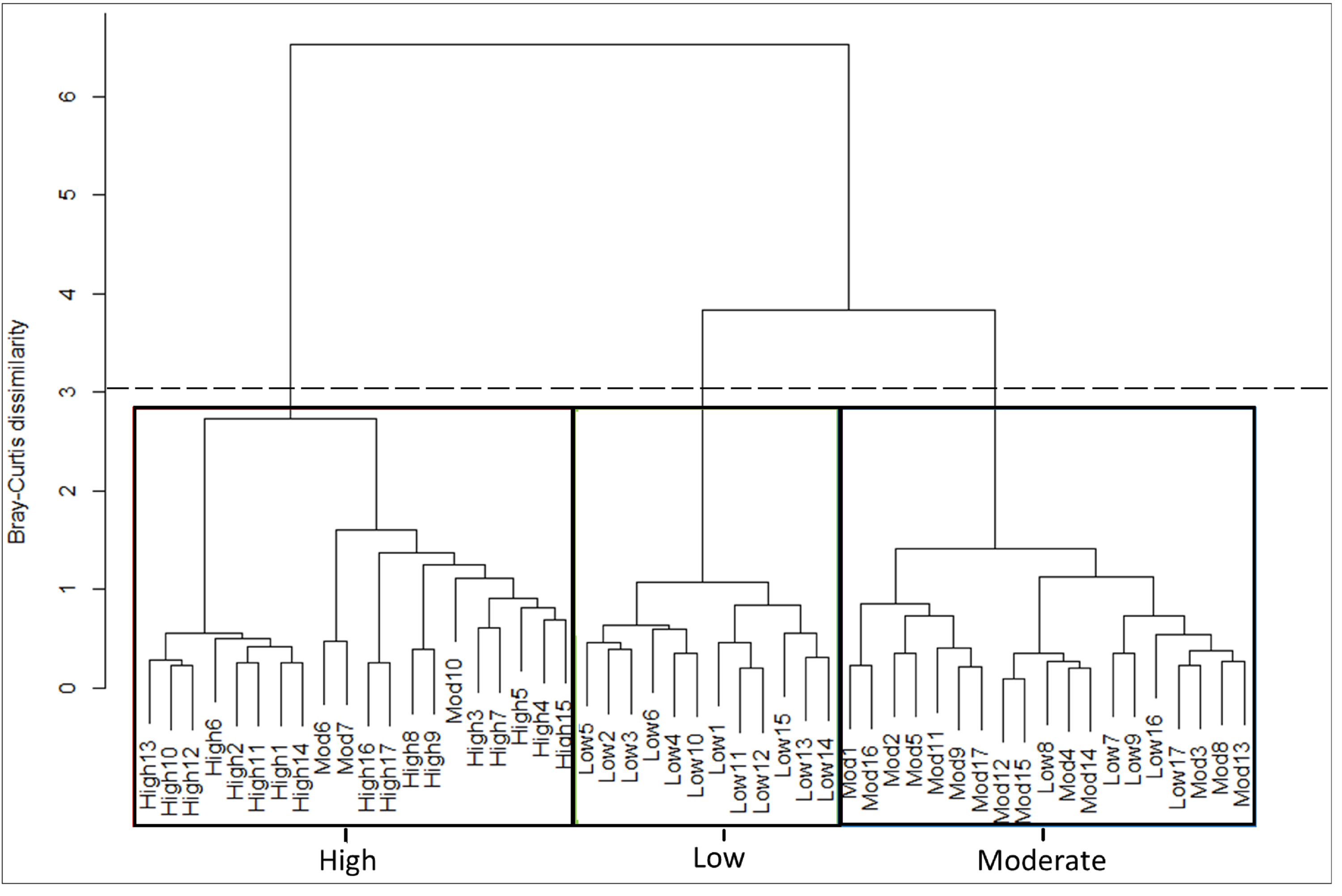

Supplement: Supplemental Information 6 — Low 1–17 = low fire exposure transects; Mod 1–17 = moderate fire- exposure transects; High 1–17 = high fire exposure transects. The dashed line indicates where the dendrogram was cut (linkage distance = 5). The rectangles delineate the three architectural guild cover abundance between three fire exposure categories. [file peerj-10-14310-s006.png]

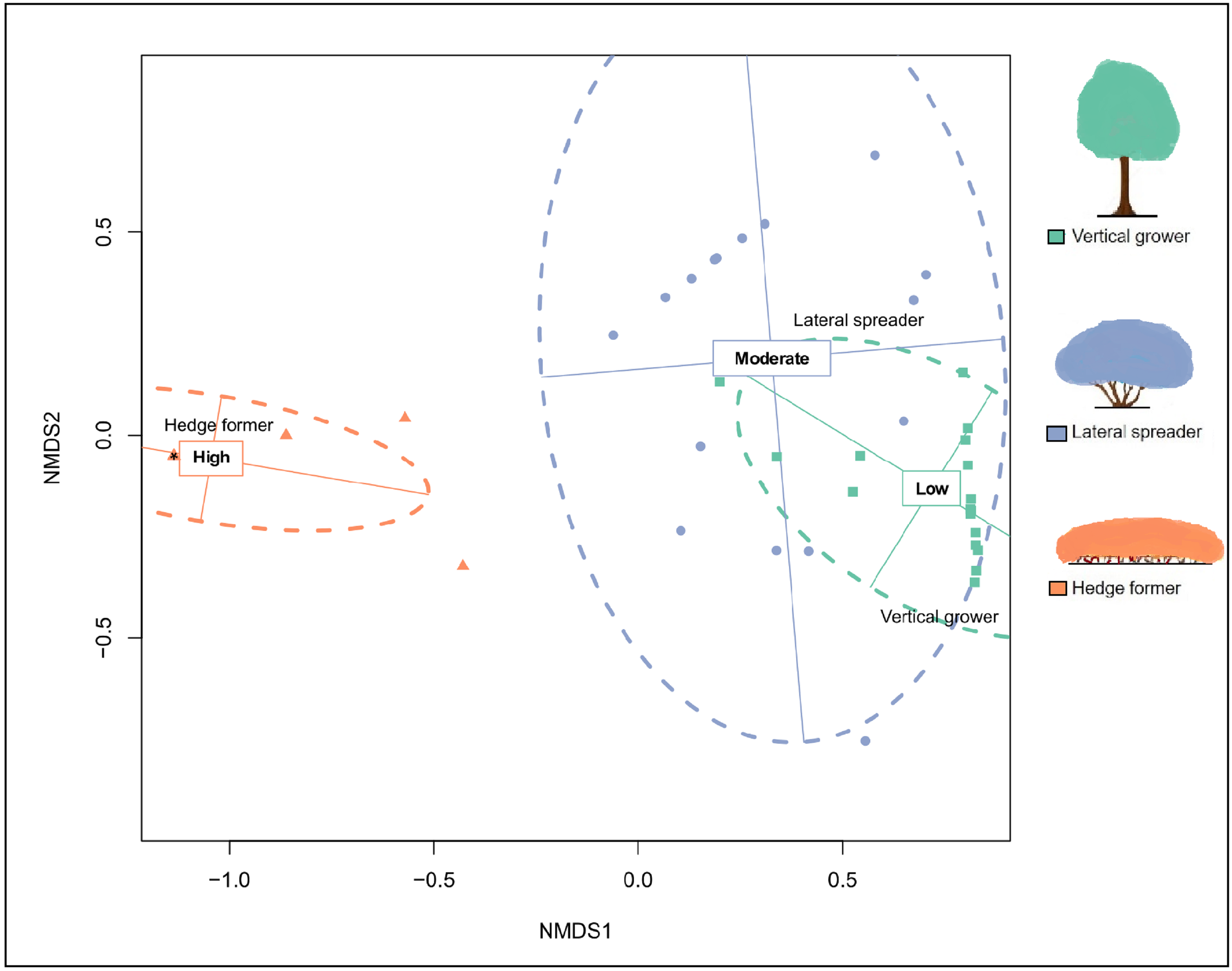

Supplement: Supplemental Information 7 — The ordination was plotted with the use of the Bray-Curtis dissimilarity matrix based on cover abundance data of architectural guilds and projected onto two-dimensional space. The shapes (triangle, square and circle) are transects colored according to their location in predetermined fire exposure categories (green = low fire exposure; blue = moderate fire exposure; orange = high fire exposure) and the cross dashed ellipses indicate 95% confidence intervals for these categories. Because of extreme structural homogeneity in high fire exposure, 14 of 17 transects plotted on top each other (indicated by an asterisk in the triangle). NMDS solution: k = 2, stress = 1.4%, linear R2 = 0.999, non-metric R2 = 1. NMDS axis 1 reflects the fire exposure gradient, which is aligned along the axis of most variation in architectural guild cover abundance. The legend (color coded) shows the architectural guild which is most common in the respective fire exposure categories. [file peerj-10-14310-s007.png]

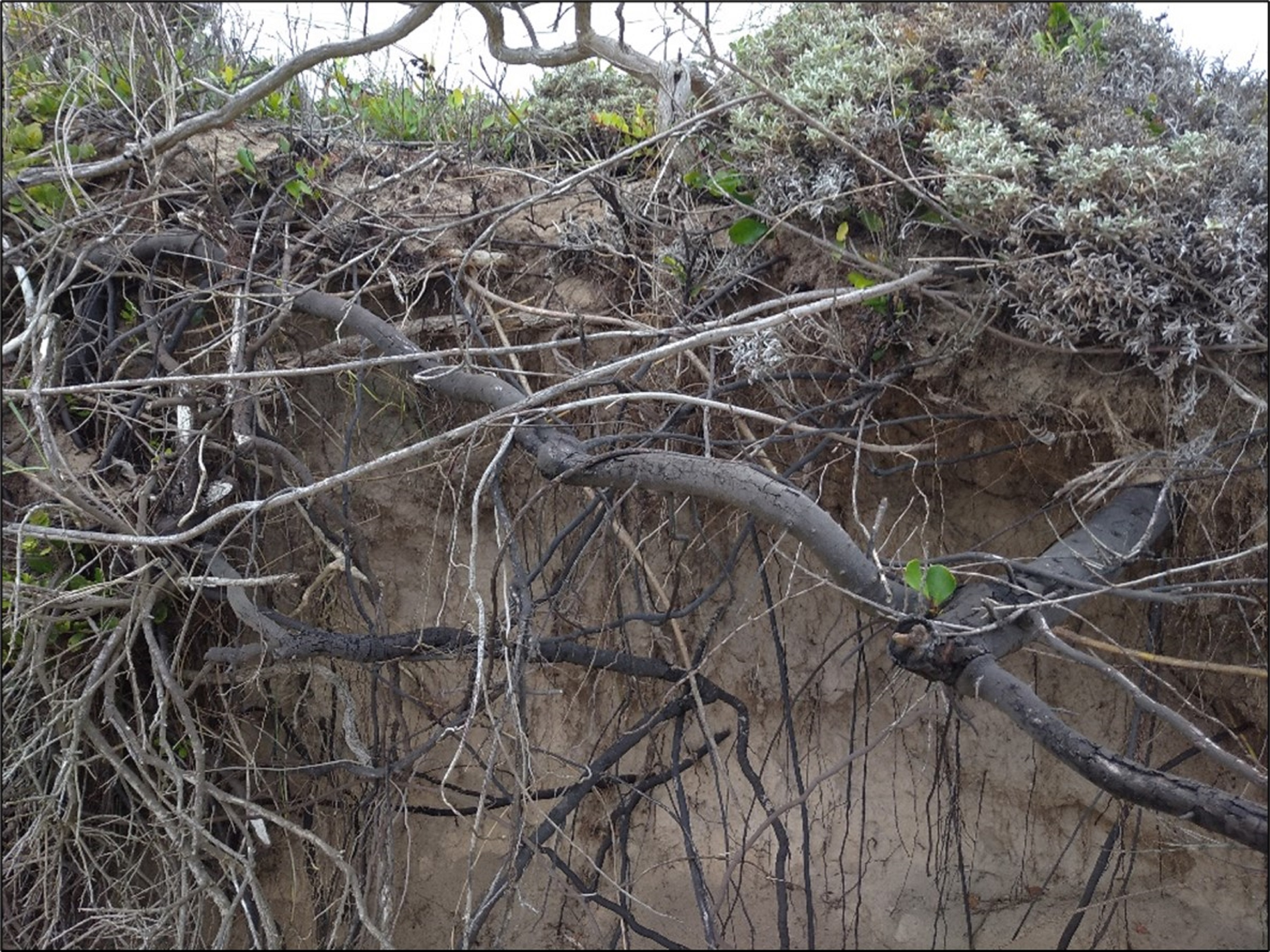

Supplement: Supplemental Information 8 [file peerj-10-14310-s008.jpg]

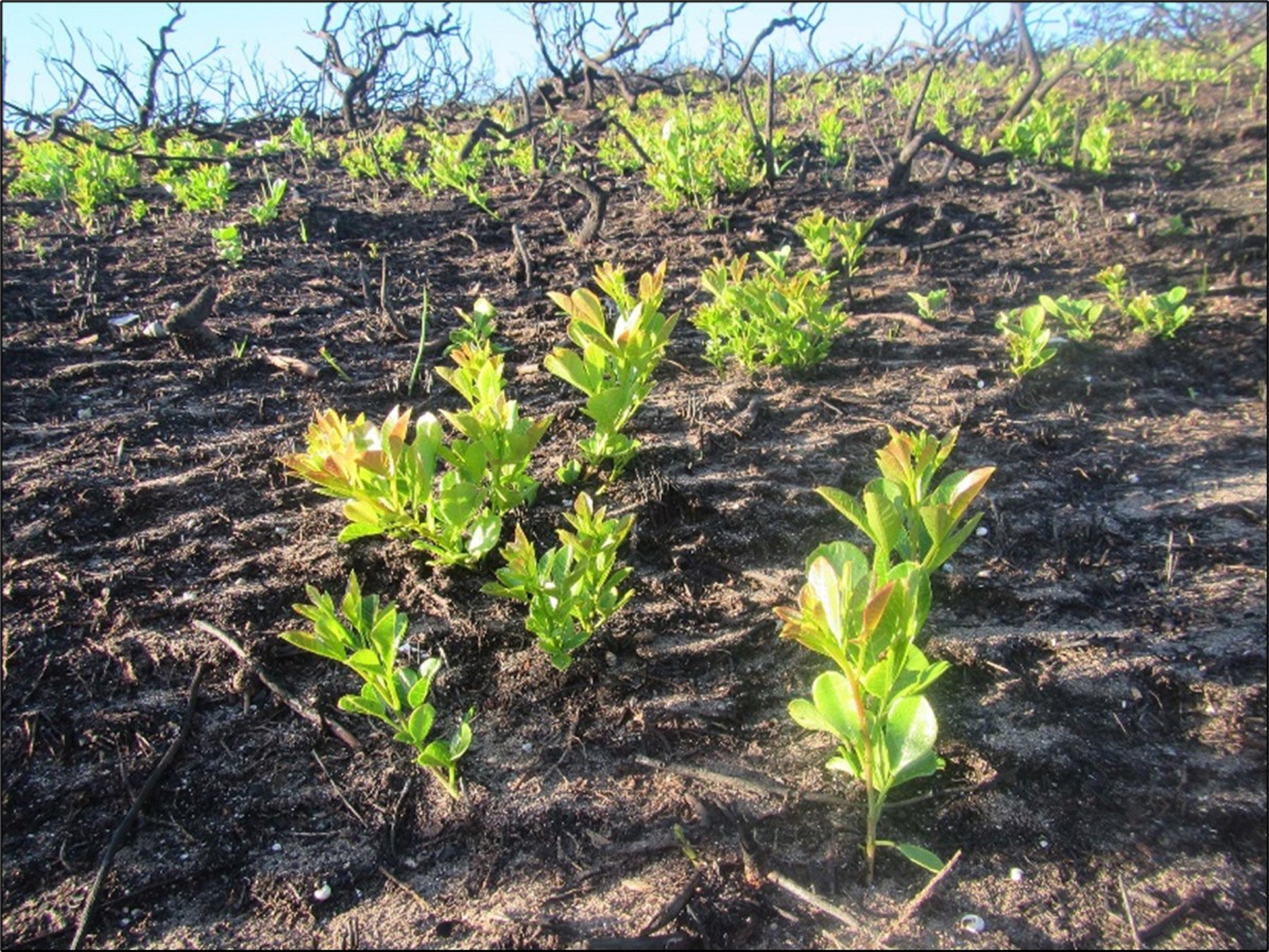

Supplement: Supplemental Information 9 [file peerj-10-14310-s009.jpg]

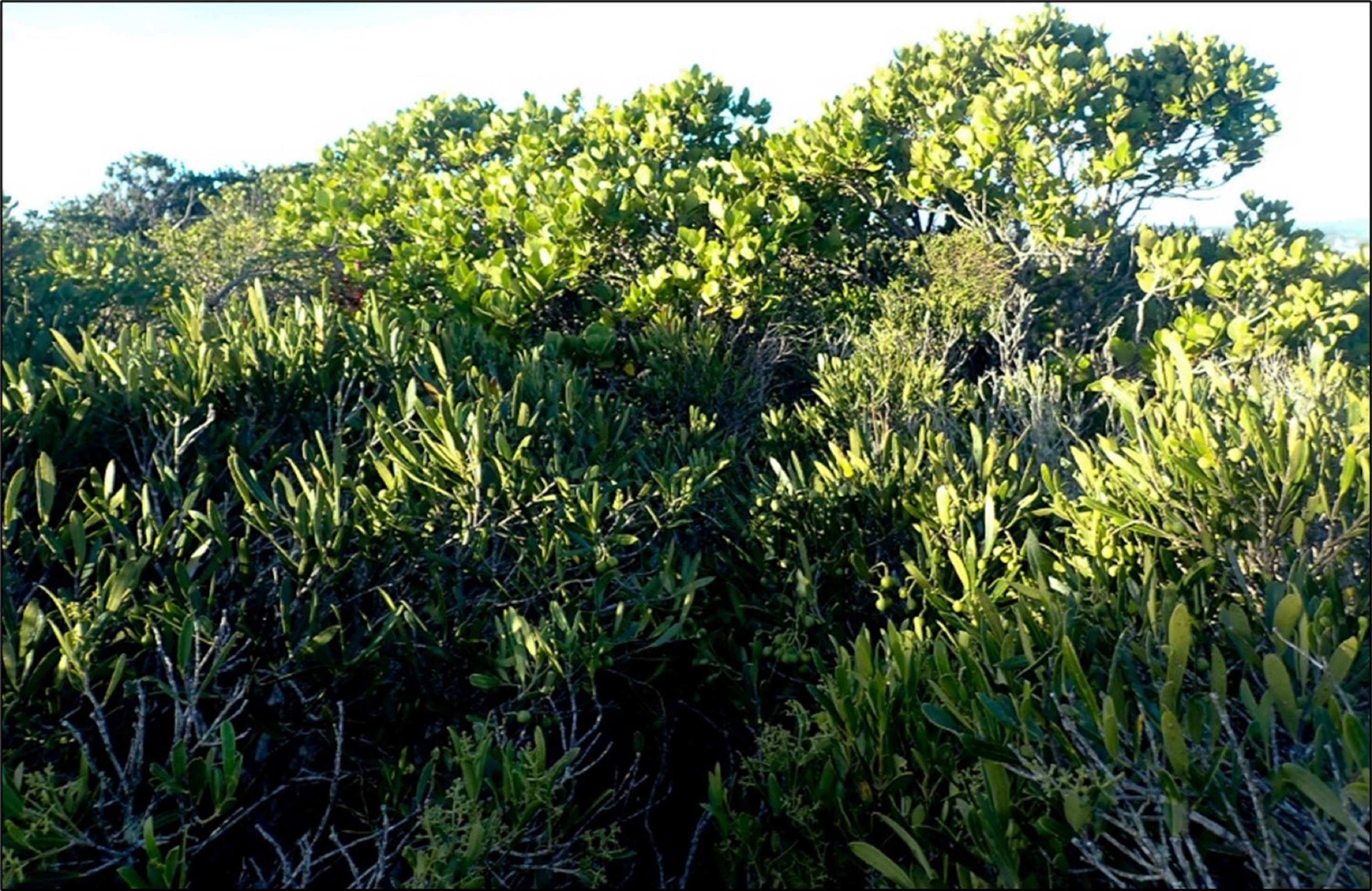

Supplement: Supplemental Information 10 [file peerj-10-14310-s010.jpg]

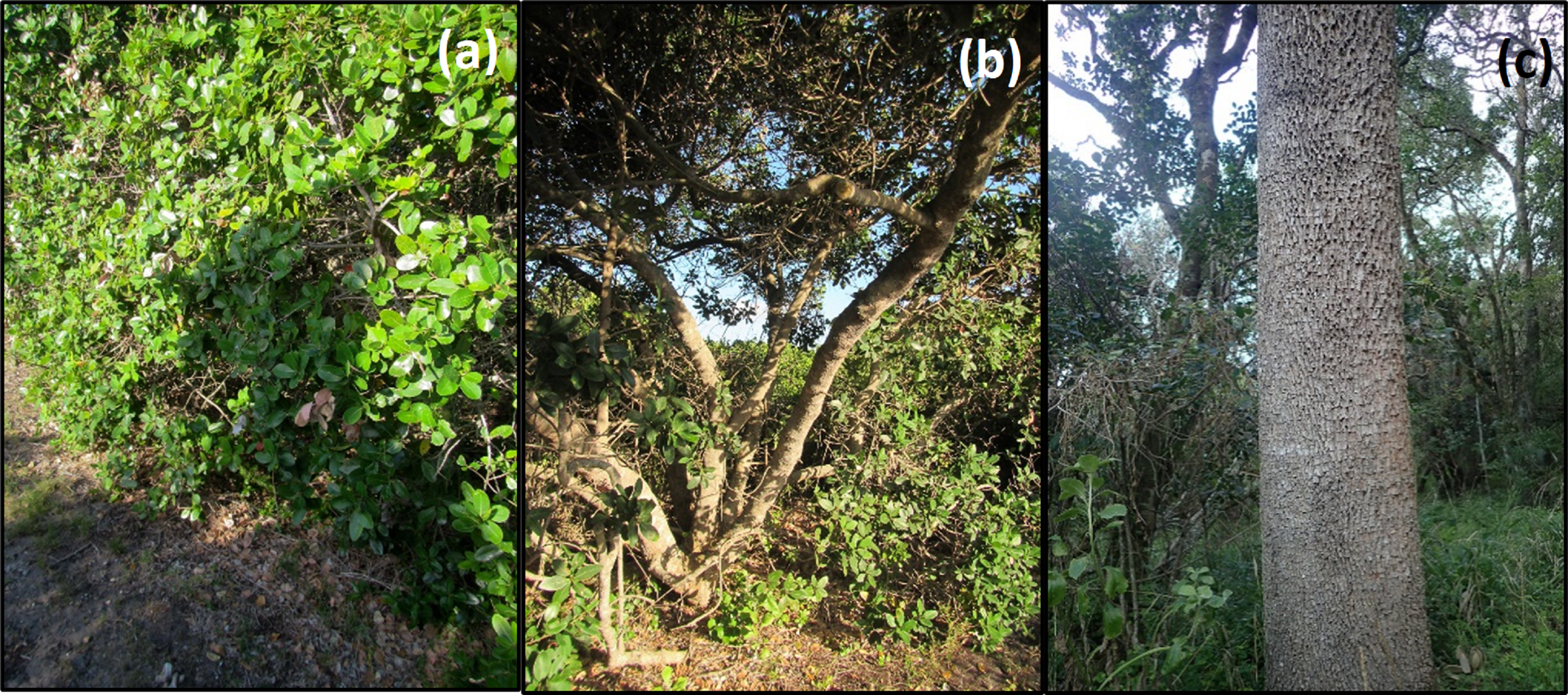

Supplement: Supplemental Information 11 [file peerj-10-14310-s011.png]
