# Supplementary material for: Canopy plant composition and structure of Cape subtropical dune thicket are predicted by the levels of fire exposure
Source: PeerJ. 2022 Nov 8;10:e14310. doi: 10.7717/peerj.14310 (PMC9651048; doi:10.7717/peerj.14310)
Supplement: Supplemental Information 12 [file peerj-10-14310-s012.docx]

| Occurrence of fire | Year | Aerial imagery | Anecdotal accounts |
| --- | --- | --- | --- |
| Yes | 1869 |  | St Francis kromme Enviro. Online Available at:https://stfranciskrommetrust.co.za/coastal  -treasure/history-2/. [Accessed 11 June 2022]. |
| None | 1961 | Aerial photo |  |
| None | 1969 | Aerial photo |  |
| Yes | 1982 |  | Mr John Booysen. Personal communication |
| None | 1985 | Aerial photo |  |
| None | 1996 |  |  |
| Yes | 1988 |  | St Francis kromme Enviro. Online Available at:https://stfranciskrommetrust.co.za/coastal  -treasure/history-2/. [Accessed 11 June 2022]. |
| Yes | 1996 |  | Mr Jan Riegaard. Personal communication  Prof. Richard Cowling. Personal communication |
| None | 2000 | Aerial photo |  |
| None | 2006 | Satellite image |  |
| None | 2009 | Satellite image |  |
| None | 2011 | Satellite image |  |
| None | 2013 | Satellite image |  |
| Yes | 2016 | Satellite image | TimesLive. Online Available at: https://www.timeslive.co.za/news /south-  africa/2016-01-26-cape-st-francis-fire-  reignites/. [Accessed 11 June 2022].  Mr Jal Riegaard. Personal communication  Prof. Richard Cowling. Personal observation |
| None | 2019 | Aerial photo (satellite) |  |

**Supplemental Table 1**: Summary of anecdotal accounts and aerial imagery consulted to establish a history of fire occurrence in and around Cape St Francis.

Notes:

Prof. Richard Cowling has extensive knowledge of the area as he started research in the St Francis area in 1979 where he did field work and lived in St Francis Bay for one year. He returned in 1981 and lived there until 1983. He returned in 1984 and lived at Cape St Francis until 1986 when he moved to Cape Town. While he was working for the University of Cape Town (1987-2000) he spent more than a month every year at Cape St Francis. In the mid 2000 he moved to Cape St Francis permanently.
