# Supplementary material for: Canopy plant composition and structure of Cape subtropical dune thicket are predicted by the levels of fire exposure
Source: PeerJ. 2022 Nov 8;10:e14310. doi: 10.7717/peerj.14310 (PMC9651048; doi:10.7717/peerj.14310)
Supplement: Supplemental Information 13 [file peerj-10-14310-s013.docx]

| **Transect** | **Slope** | **Aspect** | **Soil type** |
| --- | --- | --- | --- |
| L1 | Gentle | NNW | Deep, alkaline, moderately fertile sand |
| L2 | Moderate | NNW | Deep, alkaline, moderately fertile sand |
| L3 | Steep | NNW | Deep, alkaline, moderately fertile sand |
| L4 | Steep | S | Deep, alkaline, moderately fertile sand |
| L5 | Moderate | S | Deep, alkaline, moderately fertile sand |
| L6 | Moderate | S | Deep, alkaline, moderately fertile sand |
| L7 | Moderate | S | Deep, alkaline, moderately fertile sand |
| L8 | Steep | S | Deep, alkaline, moderately fertile sand |
| L9 | Moderate | S | Deep, alkaline, moderately fertile sand |
| L10 | Moderate | S | Deep, alkaline, moderately fertile sand |
| L11 | Gentle | NNW | Deep, alkaline, moderately fertile sand |
| L12 | Gentle | NNW | Deep, alkaline, moderately fertile sand |
| L13 | Gentle | NNW | Deep, alkaline, moderately fertile sand |
| L14 | Gentle | S | Deep, alkaline, moderately fertile sand |
| L15 | Gentle | S | Deep, alkaline, moderately fertile sand |
| L16 | Moderate | S | Deep, alkaline, moderately fertile sand |
| L17 | Gentle | NNW | Deep, alkaline, moderately fertile sand |
| M1 | Moderate | S | Deep, alkaline, moderately fertile sand |
| M2 | Gentle | S | Deep, alkaline, moderately fertile sand |
| M3 | Flat | S | Deep, alkaline, moderately fertile sand |
| M4 | Moderate | S | Deep, alkaline, moderately fertile sand |
| M5 | Gentle | S | Deep, alkaline, moderately fertile sand |
| M6 | Gentle | NNW | Deep, alkaline, moderately fertile sand |
| M7 | Flat | NNW | Deep, alkaline, moderately fertile sand |
| M8 | Steep | NNW | Deep, alkaline, moderately fertile sand |
| M9 | Steep | NNW | Deep, alkaline, moderately fertile sand |
| M10 | Moderate | S | Deep, alkaline, moderately fertile sand |
| M11 | Flat | S | Deep, alkaline, moderately fertile sand |
| M12 | Gentle | S | Deep, alkaline, moderately fertile sand |
| M13 | Moderate | NNW | Deep, alkaline, moderately fertile sand |
| M14 | Moderate | NNW | Deep, alkaline, moderately fertile sand |
| M15 | Flat | S | Deep, alkaline, moderately fertile sand |
| M16 | Moderate | S | Deep, alkaline, moderately fertile sand |
| M17 | Gentle | NNW | Deep, alkaline, moderately fertile sand |
| H1 | Gentle | NW | Deep, alkaline, moderately fertile sand |
| H2 | Gentle | S | Deep, alkaline, moderately fertile sand |
| H3 | Gentle | S | Deep, alkaline, moderately fertile sand |
| H4 | Moderate | NNW | Deep, alkaline, moderately fertile sand |
| H5 | Moderate | NNW | Deep, alkaline, moderately fertile sand |
| H6 | Gentle | NNW | Deep, alkaline, moderately fertile sand |
| H7 | Moderate | NNW | Deep, alkaline, moderately fertile sand |
| H8 | Gentle | NNW | Deep, alkaline, moderately fertile sand |
| H9 | Gentle | SSE | Deep, alkaline, moderately fertile sand |
| H10 | Gentle | SSE | Deep, alkaline, moderately fertile sand |
| H11 | Moderate | S | Deep, alkaline, moderately fertile sand |
| H12 | Flat | S | Deep, alkaline, moderately fertile sand |
| H13 | Steep | NNW | Deep, alkaline, moderately fertile sand |
| H14 | Steep | S | Deep, alkaline, moderately fertile sand |
| H15 | Gentle | NW | Deep, alkaline, moderately fertile sand |
| H16 | Gentle | S | Deep, alkaline, moderately fertile sand |
| H17 | Moderate | S | Deep, alkaline, moderately fertile sand |

**Supplemental Table 2:** The slope, aspect and soil type of if the transects located in the different fire exposure categories (L1-17, low fire exposure; M1-17, moderate fire exposure; H1-17, high fire exposure).
