# Supplementary material for: Canopy plant composition and structure of Cape subtropical dune thicket are predicted by the levels of fire exposure
Source: PeerJ. 2022 Nov 8;10:e14310. doi: 10.7717/peerj.14310 (PMC9651048; doi:10.7717/peerj.14310)
Supplement: Supplemental Information 14 — Permutational Multivariate Analysis of Variance shows that there is a significant difference between the three fire exposure categories (F = 15.051, P = 0.001). Pairwise multilevel comparison shows that all fire exposure categories are significantly different from each other (adjusted P = 0.003 for all comparisons). [file peerj-10-14310-s014.docx]

**Supplemental Table 3:** ADONIS analysis for cover abundance of dune thicket species across three fire-exposure categories.

**ADONIS (Species)**

**Df SumsOfSqs MeanSqs F.Model R2 Pr(>F)**

Fireexposure 2 6.474 3.237 15.051 0.385 0.001 ***

Residuals 48 10.323 0.215 0.615

Total 50 16.797 1.000

Permutational Multivariate Analysis of Variance shows that there is a significant difference between the three fire exposure categories (F = 15.051, P = 0.001).

**Df SumsOfSqs F.Model R2 p.value p.adjusted**

Low vs Moderate 1 1.707556 9.081 0.221 0.001 0.003 *

Low vs High 1 4.129395 18.533 0.367 0.001 0.003 *

Moderate vs High 1 3.873969 16.529 0.341 0.001 0.003 *

Pairwise multilevel comparison shows that all fire exposure categories are significantly different from each other (adjusted P = 0.003 for all comparisons).
