# Supplementary material for: Canopy plant composition and structure of Cape subtropical dune thicket are predicted by the levels of fire exposure
Source: PeerJ. 2022 Nov 8;10:e14310. doi: 10.7717/peerj.14310 (PMC9651048; doi:10.7717/peerj.14310)
Supplement: Supplemental Information 15 — Permutational Multivariate Analysis of Variance shows that there is a significant difference between the three fire exposure categories (F = 59.237, P = 0.001). Pairwise multilevel comparison shows that all fire exposure categories are significantly different from each other (adjusted P = 0.003 for all comparisons). [file peerj-10-14310-s015.docx]

**Supplemental Table 4:** ADONIS analysis for cover abundance of three architectural guilds across three fire-exposure categories.

**ADONIS**

**Df SumsOfSqs MeanSqs F.Model R2 Pr(>F)**

Fire_exposure 2 8.942 4.471 59.237 0.712 0.001 ***

Residuals 48 3.623 0.076 0.288

Total 50 12.565 1.000

Permutational Multivariate Analysis of Variance shows that there is a significant difference between the three fire exposure categories (F = 59.237, P = 0.001).

**Df SumsOfSqs F.Model R2 p.value p.adjusted**

Low vs Moderate 1 5.157 55.734 0.635 0.001 0.003 *

Low vs High 1 7.235 230.098 0.878 0.001 0.003 *

Moderate vs High 1 1.021 9.966 0.237 0.001 0.003 *

Pairwise multilevel comparison shows that all fire exposure categories are significantly different from each other (adjusted P = 0.003 for all comparisons).
